# Cardiovascular-kidney-metabolic progression associated with major adverse liver outcomes: mediating roles of plasma metabolites

**DOI:** 10.3389/fnut.2025.1675899

**Published:** 2025-10-20

**Authors:** Jingjing Liang, Yongqi Liang, Liwen Chen, Mengyi Cai, Shuxian Li, Lige He, Yining Xu, Yilan Tan, Linna Li, Xianbo Wu, Mengchen Zou

**Affiliations:** ^1^Department of Endocrinology and Metabolism, Nanfang Hospital, Southern Medical University, Guangzhou, China; ^2^Department of Epidemiology, School of Public Health, Southern Medical University, Guangzhou, China; ^3^Department of Health Care, Jiangmen Maternity and Child Health Care Hospital, Jiangmen, China; ^4^Department of Respiratory and Critical Care Medicine, Nanfang Hospital, Southern Medical University, Guangzhou, China; ^5^School of Public Health, Southern Medical University, Guangzhou, China; ^6^Department of Epidemiology, School of Public Health, Guangdong Provincial Key Laboratory of Tropical Disease Research, Southern Medical University, Guangzhou, China

**Keywords:** CKM syndrome, MASLD, major adverse liver outcomes, mortality, plasma metabolomics

## Abstract

**Objectives:**

Previous research has not yet established whether and how cardiovascular-kidney-metabolic (CKM) syndrome progression affects liver outcomes.

**Methods:**

This prospective study utilized data from the UK Biobank (UKB) cohort, including 415,713 individuals without prevalent liver diseases or substance use disorder. The CKM syndrome stages were defined according to the Presidential Advisory from the American Heart Association. Outcomes were major adverse liver outcomes (MALOs), including hospitalization for metabolic dysfunction-associated steatotic liver disease (MASLD), severe liver disease (SLD), and liver-specific mortality. Cox proportional hazards models examined the association between CKM stages and MALOs. The *CMAverse* R package was used to investigate the potential mediating effects of plasma metabolomic data.

**Results:**

After multivariable adjustment, a higher CKM stage was associated with elevated risks of incident MASLD hospitalization [hazard ratios (HRs) = 7.38, 95% confidence intervals (CIs): 4.34, 12.55], SLD hospitalization (HR = 3.46, 95% CI:1.94, 6.16), and liver-specific mortality (HR = 4.35; 95% CI: 1.38, 13.69). CKM components were, respectively, and cumulatively associated with MALOs (all *p* < 0.05). Mediation analyses indicated that tyrosine partially mediated the associations between CKM stage and MASLD-related hospitalization (7.62%), SLD-related hospitalization (9.46%), and liver-related death (11.19%), while linoleic acid-to-total fatty acids ratio partially mediated MASLD hospitalization (41.18%), SLD hospitalization (34.30%), and liver-related death (45.17%) (all *q* < 0.001).

**Conclusion:**

CKM progression elevates MALO risk, partially mediated by amino acids and fatty acids. These findings identify high-risk patients who may benefit from targeted liver surveillance for secondary prevention of CKM syndrome.

## 1 Introduction

Chronic liver disease and its subsequent complications (cirrhosis and liver cancer) contributed to significant economic burden and mortality (1). Metabolic dysfunction-associated steatotic liver disease (MASLD), renamed from non-alcoholic fatty liver disease (NAFLD), represents the most widespread chronic liver disorder (2, 3), leading to a rapidly rising inpatient clinical and economic burden (4). It has emerged as the most common contributor to cirrhosis and hepatocellular carcinoma (HCC) (2) and underlies the accelerating trend of liver-specific mortality (5). Cardiovascular-kidney-metabolic (CKM) syndrome is known as the multisystem dysfunction stemming from the inter-relatedness between metabolic risk factors (including diabetes and obesity), chronic kidney disease (CKD), and cardiovascular disease (CVD). Its conceptualization emphasizes the need for the multidisciplinary management of these dynamically progressive and interactive diseases to prevent the adverse consequences rather than focusing on individual diseases (6, 7).

MASLD and CKM syndrome share overlapping pathophysiologic processes, including lipid toxicity, inflammation, and insulin resistance (7, 8). In addition, CKM components, such as diabetes and obesity, have a great impact on the development of MASLD, cirrhosis, and HCC (8). However, previous research almost exclusively focused on the association between individual CKM components and MASLD (9, 10), liver complications (10, 11), and mortality (10) rather than evaluating these components as an integrated and progressive syndrome. It therefore remains unclear whether and how CKM syndrome stage affects liver outcomes. Furthermore, MASLD exhibits marked heterogeneity in progression rates and clinical outcomes; the majority of patients exhibit stable or slow progression without developing cirrhosis or liver-related mortality (2). Given this heterogeneity, identifying high-risk individuals prone to progressing to MASLD-related hospitalization is imperative to reduce healthcare expenditures. Therefore, this study primarily focused on severe MASLD and other liver-related outcomes requiring hospitalization. Using a prospective longitudinal design, we examined the risk of major adverse liver outcomes (MALOs), including MASLD hospitalization, severe liver disease (SLD) hospitalization, and liver-specific mortality, across different CKM syndrome stages.

Plasma metabolites, especially free fatty acids (FFAs) and amino acids, have been identified as promising risk factors for MASLD and adverse liver complications (12, 13). In addition, previous research revealed that several CKM components, including diabetes and obesity, may alter free fatty acids and branched-chain amino acids (BCAAs) by insulin resistance and systemic inflammation (14, 15), which were key pathological processes shared by CKM syndrome and MALOs. Therefore, we hypothesized that these metabolites may mediate the association between CKM stages and MALOs.

## 2 Methods

### 2.1 Study population

More than 500,000 participants aged 37–73 years were prospectively recruited by the UK Biobank cohort study throughout the UK from 2006 to 2010. The baseline visit included biological samples, lifestyle questionnaires, physical measurements, and individual medical history. Plasma samples collected at baseline were randomly selected from approximately 280,000 UK Biobank participants for nuclear magnetic resonance (NMR) metabolomic measurements. Written informed consent was obtained from every participant. The UK North West Multicenter Research Ethics Committee reviewed and approved the protocol of the UK Biobank.

**Figure 1:**
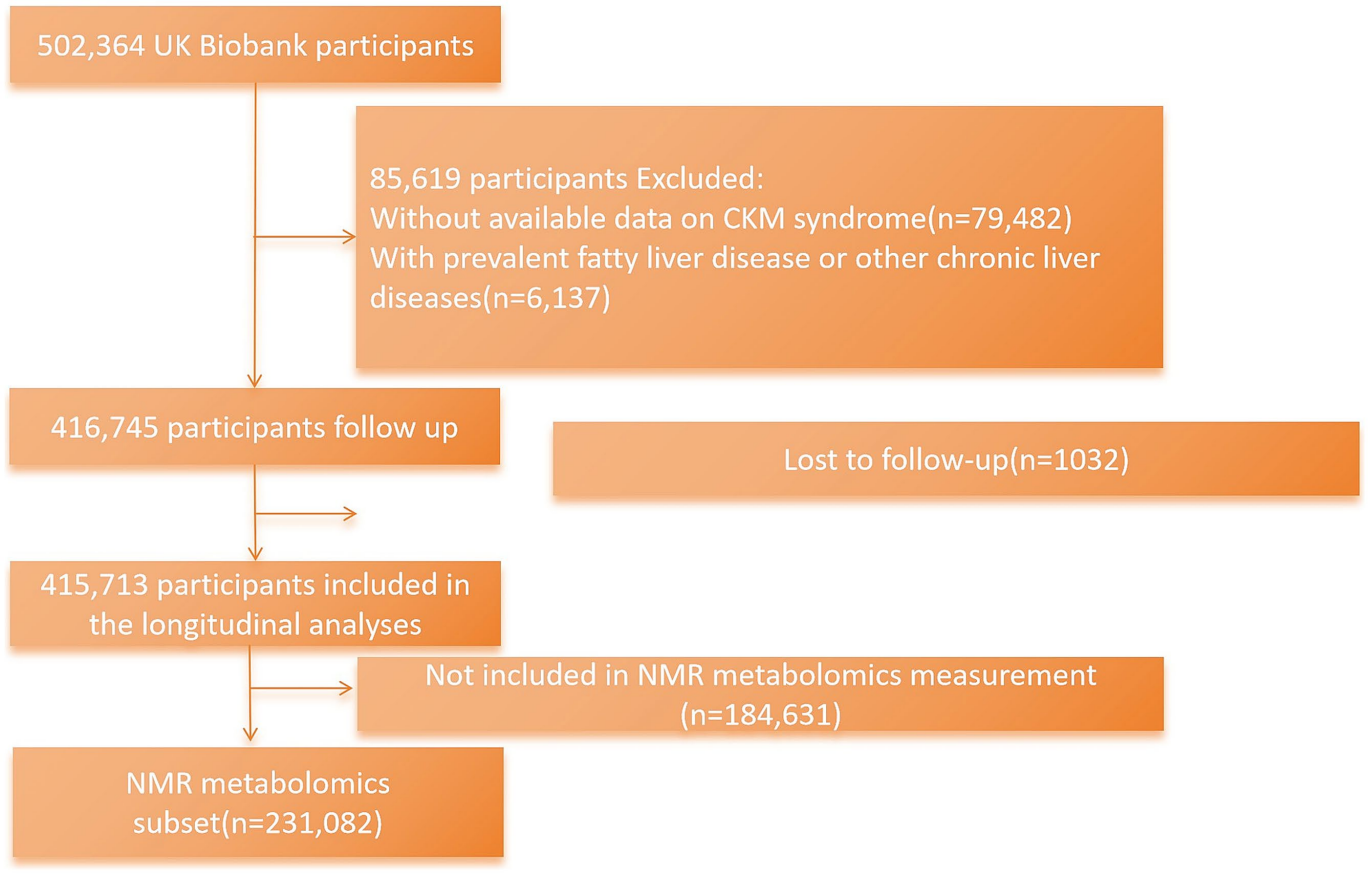
Flowchart of enrollment.

The current research excluded participants who had missing available data necessary for defining CKM syndrome stage (n = 79,482); those with prevalent MASLD, other chronic liver diseases, or substance use disorder at baseline (n = 6,137) (International Statistical Classification of Diseases and Related Health Problems, 10th revision (ICD-10) codes for these diseases as the exclusion criteria are shown in Supplementary Table S3); and participants who were lost to follow-up (n = 1,032). Finally, there were 415,713 UK Biobank individuals included in the main analyses. Participants with NMR metabolomic data were further included in the mediation analysis (n = 231,082) (Figure 1).

### 2.2 Assessment of CKM syndrome stage

CKM syndrome was conceptualized as the multisystem dysfunction deriving from the interrelatedness of metabolic, CKD, and CVD risk factors (6, 7). A 5-stage system was established for further classification: (1) stage 0: no CKM syndrome risk factors emerge; (2) stage 1: prediabetes or obesity; (3) stage 2: the presence of metabolic syndrome [MetS], diabetes, hypertriglyceridemia, hypertension, or moderate to high risk for CKD; (4) stage 3: very high risk for CKD or high risk for CVD; and (5) stage 4: cardiovascular diseases accompanied by other CKM syndrome risk factors.

Prediabetes was identified in individuals with a glycated hemoglobin (HbA1c) of 5.7–6.4% (39–47 mmol/mol) or with impaired fasting glucose (IFG, fasting glucose 6.1–7.0 mmol/L) (16). The CKD risk was defined according to Kidney Disease: Improving Global Outcomes (KDIGO) methods (17). An updated CKM risk algorithm was used to calculate predicted 10-year CVD risk, which incorporates age, sex, body mass index (BMI), blood pressure (BP), diabetes, cholesterol, antihypertensive use, statin use, tobacco use, and estimated glomerular filtration rate (eGFR) in the model (18, 19). Subclinical CVD was captured based on either a ≥ 20% 10-year CVD risk or high CKD risk status. Detailed definitions of CKM syndrome traits are shown in Supplementary Tables S1, S2 (20).

### 2.3 Assessment of outcomes

In the UK Biobank (UKB), health-related outcomes were captured through National Health Service records. Incident hospitalization for MASLD is defined by the first hospitalization (UKB code 41270) owing to MASLD, according to guidance on examining MASLD in electronic healthcare record-based research (Supplementary Table S4) (21). Specifically, it was identified using established ICD-10 codes (K76.0, K75.8) (21–23). Secondary outcomes were severe liver disease (defined as the presence of at least one of the following conditions: cirrhosis [K74.0, K74.1, K74.2, K74.6, K70.2, K70.3, K70.4, K76.6, and I85], HCC [C22.0], intrahepatic cholangiocarcinoma [C22.1], and other liver cancers [C22.2-C22.4, C22.7, and C22.9]) and liver-specific mortality (K70.2, K70.3, K70.4, K74.0, K74.1, K74.2, K74.6, K75.8, K76.0, K76.6, I85, C22.0, C22.1, C22.2-C22.4, C22.7, and C22.9) (23–25). Every participant in the current study was followed from baseline until the earliest event of death, occurrence of outcomes, or last available data collection (20 July 2021).

### 2.4 Assessment of covariates and metabolic biomarkers

The covariates in this study were included based on previous knowledge and the directed acyclic graph (Supplementary Figure S1). All participants in the UKB underwent verbal interviews and self-reported touchscreen questionnaires, as well as physical measurements to collect information on sociodemographic (sex, age, ethnicity, and education level), area-based social deprivation (Townsend deprivation index, TDI), and lifestyle (duration of sleep, intensity of physical activity, food intake, smoking status, and drinking status). Dietary risk was quantified using an established cumulative score [cumulative dietary risk factor score (CDRFS)] with a 10-point scale, where 0 represented the most healthful dietary characteristics and 9 signified the least healthful dietary profile. It was derived from the intake of nine common foods (comprising meat consumption [red meat and processed meat], spread, milk, fish, cereal, water, salt, and vegetables, along with fruits) based on the UK guidelines (26).

NMR metabolomics was quantified in randomly selected plasma samples using a high-throughput NMR-based metabolomic platform developed by Nightingale Health Ltd. The metabolic biomarkers of interest include a total of 10 types of amino acids (alanine, glutamine, glycine, histidine, isoleucine, leucine, valine, phenylalanine, tyrosine, and total branched-chain amino acids) and 17 types of FFA biomarkers (total fatty acid [TFA], omega-3 fatty acid, omega-6 fatty acid, polyunsaturated fatty acid [PUFA], monounsaturated fatty acid [MUFA], saturated fatty acid [SFA], linoleic acid, docosahexaenoic acid [DHA], omega-3 fatty acid-to-total fatty acid percentage [omega-3/TFA], omega-6 fatty acid-to-TFA percentage [omega-6/TFA], PUFA-to-TFA percentage [PUFA/TFA], MUFA-to-TFA percentage [MUFA/TFA], SFA-to-TFA percentage [SFA/TFA], linoleic acid-to-TFA percentage [LA/TFA], DHA-to-TFA percentage [DHA/TFA], PUFA-to-MUFA ratio [PUFA/MUFA], and omega-6 fatty acid-to-omega-3 fatty acid ratio [omega-6/omega-3]) measured in the UK Biobank. Additional information on covariate measurement and biological samples is detailed on the UKB website.^1^

### 2.5 Statistical analysis

Participant baseline characteristics were reported as count (percentage) for categorical variables, while they were reported as mean and standard deviation (SD) for continuous variables. Baseline characteristics across different CKM syndrome stages were compared using the Kruskal–Wallis, chi-squared, or ANOVA test, whichever was appropriate.

The associations between CKM stages and the risk of MALOs were quantified using multivariate Cox proportional hazards models. Assessment of Schoenfeld residuals detected no evidence of proportional hazards assumption violation. For linear trend analysis, CKM stages were modeled as an ordered categorical variable. Two multivariate models were built. Multivariate model 1 in the current study was adjusted for sex and age, ethnic background (white or other), education level (higher education or other), and TDI (continuous), and model 2 was additionally adjusted for sleep duration (< 7 h, 7 h to ≤ 9 h, > 9 h), physical activity (low, moderate, and high), CDRFS (continuous), smoking status (never, previous, and current), and drinking status (never, previous, and current). For variables with missing data, categorical covariates were assigned to a separate category, and continuous variables were imputed using sex-specific means. The variance inflation factor test detected no evidence of multicollinearity among different covariates. Additionally, the associations of individual and cumulative CKM syndrome traits with incident hospitalization for MALOs were additionally estimated using Cox proportional hazards models. In mediation analyses, the *CMAverse* R package was used to investigate a potential mediating effect of interest metabolic biomarkers on the associations between CKM stages and MALOs. The Benjamini–Hochberg method, which controls the false discovery rate (FDR), was used to adjust for multiple testing, with significance defined as an adjusted *p*-value (q-value) of < 0.05.

Stratified analyses were carried out by sex, age (< 60 or ≥ 60 years), TDI (lower and higher), physical activity (moderate, high, or other), CDRFS (tertiles 1 to 3), sleep duration (ideal sleep duration [7 h to ≤ 9 h] or other), smoking status (current or other), and drinking status (current or other) to explore possible modifications of the associations between CKM stage and study outcomes. Multiplicative interactions were further calculated by inserting the product terms into the fully adjusted Cox models. Stratified analysis for each stratifying variable was performed with adjustment for all covariates except the variable used for stratification. Sensitivity analyses were further carried out: (1) considering death as a competing risk (in terms of liver-related mortality, non-liver-specific mortality was treated as a competing risk); (2) using inverse probability of treatment weighting (IPTW); (3) using the QRISK 3 score to predict the 10-year risk of cardiovascular disease (27); (4) eliminating individuals who suffered from incident MALOs during the first 2 years to control reverse causation bias; (5) extending landmark analyses by 5 and 7 years to investigate the relationship between CKM and long-term risks of MALOs; and (6) imputed missing covariate data with multiple imputations with chained equation.

Stata version 18.0 and R version 4.3.2 were used in data analysis. A two-tailed *p-value* of < 0.05 was considered statistically significant.

## 3 Results

### 3.1 Baseline characteristics

Baseline characteristics stratified by CKM syndrome stages are shown in Table 1. Among 415,713 individuals aged 56.56 ± 8.08 years on average in the current study, 46.2% (191,932) of the participants were men, 94.5% (392,741) were white, and 32.3% (134,483) had a college or university degree. Participants at stage 4, compared with those at stage 0, were less likely to have an ideal sleep duration (66.9% versus 78.0%), engage in a moderate-to-high-intensity physical activity (31.2% versus 34.4%; 28.9% versus 35.7%), or be a current alcohol user (87.7% versus 93.4%). Individuals at stage 4 were more likely to be current smokers (12.8% versus 11.9%), to have a higher CDRFS (5.09 ± 1.48 versus 5.04 ± 1.46), and to have a higher TDI (− 0.74 ± 3.35 versus–1.30 ± 3.09).

**Table 1:** Baseline characteristics of participants across CKM syndrome stages.

Characteristics	CKM syndrome stage					
	Total	Stage 0	Stage 1	Stage 2	Stage 3	Stage 4
Participants	415,713	5,172	4,313	340,528	38,505	27,195
Men, *n* (%)	191,932 (46.2)	1,308 (25.3)	1,437 (33.3)	149,143 (43.8)	21,726 (56.4)	18,318 (67.4)
Age, mean (SD)	56.56 (8.08)	52.07 (7.97)	53.65 (8.08)	55.67 (7.98)	61.75 (6.59)	61.56 (6.29)
Townsend deprivation index, mean (SD)	−1.34 (3.07)	−1.30 (3.09)	−1.11 (3.18)	−1.40 (3.03)	−1.26 (3.10)	−0.74 (3.35)
Ethnicity (%)						
White	392,741 (94.5)	4,929 (95.3)	3,948 (91.5)	321,695 (94.5)	36,397 (94.5)	25,772 (94.8)
Non-white	21,565 (5.2)	221 (4.3)	350 (8.1)	17,724 (5.2)	1,949 (5.1)	1,321 (4.9)
Missing	1,407 (0.3)	22 (0.4)	15 (0.3)	1,109 (0.3)	159 (0.4)	102 (0.4)
Education (%)						
College or university degree	134,483 (32.3)	2,341 (45.3)	1,529 (35.5)	114,927 (33.7)	9,833 (25.5)	5,853 (21.5)
Other degree	276,912 (66.6)	2,795 (54.0)	2,749 (63.7)	222,330 (65.3)	28,138 (73.1)	20,900 (76.9)
Missing	4,318 (1.0)	36 (0.7)	35 (0.8)	3,271 (1.0)	534 (1.4)	442 (1.6)
Sleep duration, *n* (%)						
*N* < 7 h	101,768 (24.5)	1,064 (20.6)	1,175 (27.2)	82,810 (24.3)	9,067 (23.5)	7,652 (28.1)
7 h > =*N* > =9 h	304,117 (73.2)	4,036 (78.0)	3,043 (70.6)	2,50,758 (73.6)	28,099 (73.0)	18,181 (66.9)
*N* > 9 h	7,359 (1.8)	56 (1.1)	74 (1.7)	5,113 (1.5)	1,043 (2.7)	1,073 (3.9)
Missing	2,469 (0.6)	16 (0.3)	21 (0.5)	1847 (0.5)	296 (0.8)	289 (1.1)
Physical activity, *n* (%)						
Low	63,056 (15.2)	712 (13.8)	670 (15.5)	50,710 (14.9)	6,011 (15.6)	4,953 (18.2)
Moderate	1,37,269 (33.0)	1,777 (34.4)	1,465 (34.0)	1,13,137 (33.2)	12,403 (32.2)	8,487 (31.2)
High	1,36,416 (32.8)	1,847 (35.7)	1,374 (31.9)	1,13,335 (33.3)	11,988 (31.1)	7,872 (28.9)
Missing	78,972 (19.0)	836 (16.2)	804 (18.6)	63,346 (18.6)	8,103 (21.0)	5,883 (21.6)
Smoking status, *n* (%)						
Never	2,27,733 (54.8)	3,178 (61.4)	2,498 (57.9)	1,97,893 (58.1)	13,424 (34.9)	10,740 (39.5)
Previous	1,44,384 (34.7)	1,381 (26.7)	1,309 (30.4)	1,08,092 (31.7)	20,615 (53.5)	12,987 (47.8)
Current	43,596 (10.5)	613 (11.9)	506 (11.7)	34,543 (10.1)	4,466 (11.6)	3,468 (12.8)
Drinking status, *n* (%)						
Never	18,023 (4.3)	175 (3.4)	216 (5.0)	14,495 (4.3)	1,585 (4.1)	1,552 (5.7)
Previous	14,270 (3.4)	157 (3.0)	137 (3.2)	10,622 (3.1)	1,603 (4.2)	1,751 (6.4)
Current	382,977 (92.1)	4,833 (93.4)	3,955 (91.7)	315,082 (92.5)	35,263 (91.6)	23,844 (87.7)
Missing	443 (0.1)	7 (0.1)	5 (0.1)	329 (0.1)	54 (0.1)	48 (0.2)
CDRPS, [mean (SD)]	5.08 (1.49)	5.04 (1.46)	5.18 (1.47)	5.07 (1.49)	5.18 (1.47)	5.09 (1.48)

Higher education indicates college and university degree, university, National Vocational Qualification, Higher National Diploma, Higher National Certificates, or equivalent; SD, standard deviation. CDRFS, cumulative dietary risk factor score.

### 3.2 Associations between different CKM stages and risk of major adverse liver outcomes

During a follow-up of 14.58 years, 4,845 participants reported incident hospitalization for MASLD, 2,626 participants were hospitalized due to severe liver disease, and 952 participants died of liver-related diseases. As shown in Table 2, participants at a higher CKM stage had a higher risk of incident MASLD hospitalization, severe liver disease hospitalization, and an elevated liver-specific mortality. After adjusting for potential confounders, such as sex, age, ethnicity, education level, TDI, sleep duration, physical activity intensity, smoking status, drinking status, and CDRFS, these associations remained significant. In model 2, in comparison with the reference group, individuals at other stages had a higher risk of incident MASLD hospitalization: in stage 1, hazard ratio (HR) = 2.64 (95% confidence interval [CI]: 1.42, 4.92); in stage 2, HR = 3.54 (95% CI: 2.09, 5.98); in stage 3, HR = 5.86 (95% CI: 3.45, 9.97); and in stage 4, HR = 7.38 (95% CI: 4.34, 12.55). After full adjustment, participants at stage 4 had a higher rate of hospitalization for severe liver disease and higher liver-specific mortality than those at stages 0: severe liver disease hospitalization (HR = 3.46; 95% CI: 1.94, 6.16) and liver-specific mortality (HR = 4.35; 95% CI: 1.38, 13.69).

**Table 2:** HRs for major adverse liver outcomes by CKM syndrome stage.

Model	CKM syndrome stage					*p*_for trend_
	Stage 0	Stage 1	Stage 2	Stage 3	Stage 4	
MASLD						
Events/subjects	14/5,172	34/4,313	3448/340,528	691/38,505	658/27,195	
Model 1	1	2.75 (1.48, 5.13)	3.63 (2.14, 6.13)	6.45 (3.79, 10.96)	8.34 (4.90, 14.18)	<0.001
Model 2	1	2.64 (1.42, 4.92)	3.54 (2.09, 5.98)	5.86 (3.45, 9.97)	7.38 (4.34, 12.55)	<0.001
Severe liver disease						
Events/subjects	12/5172	23/4313	1647/340528	515/38505	429/27195	
Model 1	1	2.02 (1.01, 4.06)	1.64 (0.93, 2.89)	3.47 (1.95, 6.17)	3.79 (2.13, 6.75)	<0.001
Model 2	1	1.96 (0.97, 3.94)	1.63 (0.93, 2.88)	3.20 (1.80, 5.69)	3.46 (1.94, 6.16)	<0.001
Liver-specific mortality						
Events/subjects	3/5,172	4/4,313	578/340,528	206/38,505	161/27,195	
Model 1	1	1.37 (0.31, 6.13)	2.13 (0.69, 6.63)	4.62 (1.47, 14.49)	4.78 (1.52, 15.04)	<0.001
Model 2	1	1.31 (0.29, 5.87)	2.13 (0.68, 6.62)	4.13 (1.32, 12.96)	4.35 (1.38, 13.69)	<0.001

*Model 1 was adjusted for age, gender, ethnic background, education, and TDI. Model 2 was additionally adjusted for sleep duration, physical activity, CDRFS, smoking status, and drinking status*.

The 10-year cumulative incidence curves were generated according to the reviewer’s kind advice. The 10-year cumulative incidence of MASLD increased significantly with higher CKM stages: stage 0 (0.16%), stage 1 (0.45%), stage 2 (0.61%), stage 3 (1.21%), and stage 4 (1.71%) (log-rank *p* < 0.001) (Supplementary Figure S2). Similarly, the highest incidence of severe liver disease and liver-specific mortality was observed in stage 4 participants (1.19% for severe liver disease and 0.43% for mortality) (Supplementary Figures S3, S4). The model for liver-specific mortality demonstrated the highest discrimination (C-index = 0.743), followed by the model for SLD (C-index = 0.709). The model for MASLD progression showed more modest discrimination (C-index = 0.658).

### 3.3 Association between individual and cumulative CKM syndrome traits and incident MALOs

**Figure 2:**
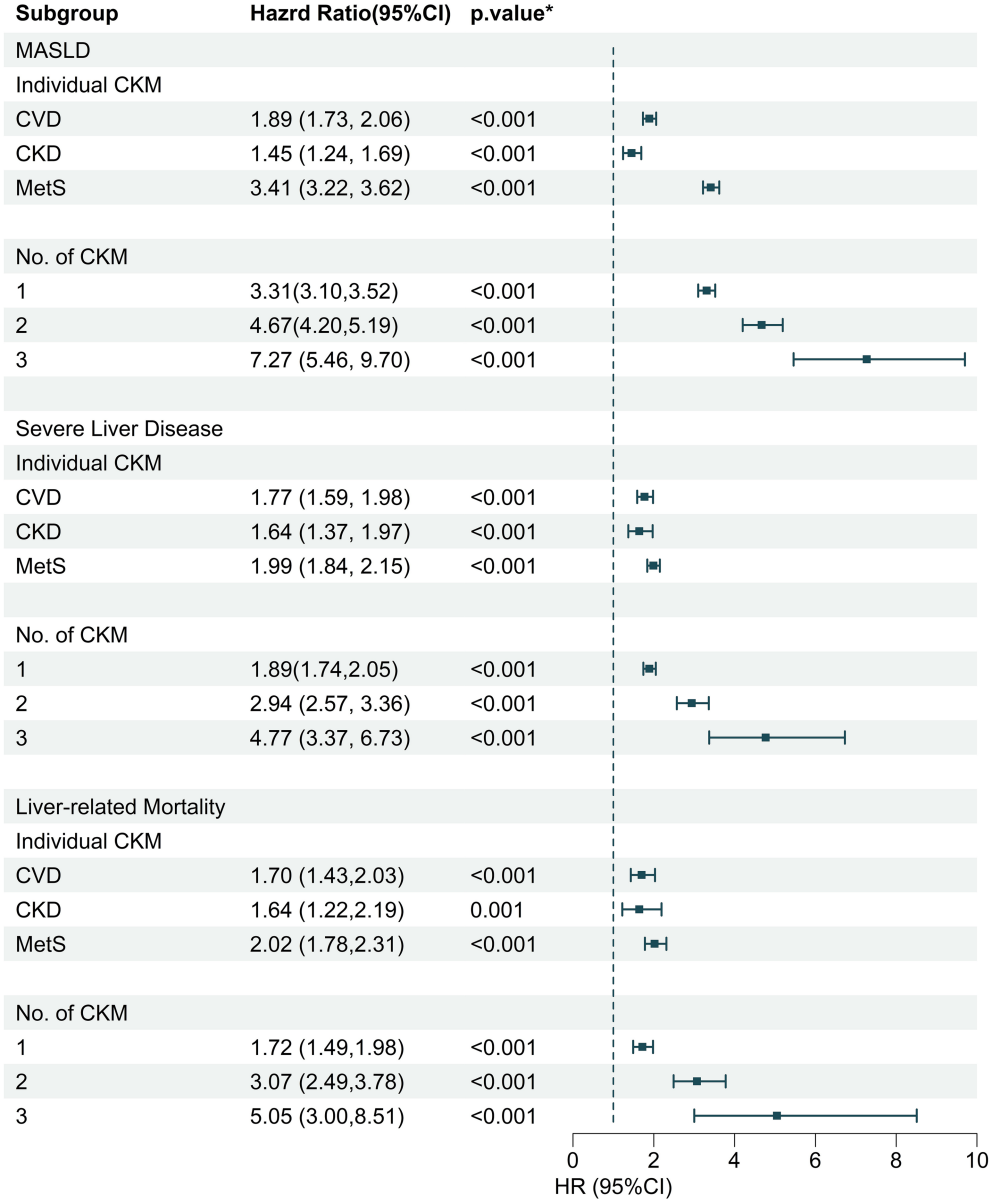
The association of individual and cumulative CKM disease numbers with incident MASLD, severe liver disease, and liver-specific mortality. CKM, cardiovascular-kidney-metabolic syndrome; MASLD, metabolic dysfunction-associated fatty liver disease; HR, hazard ratio; CI, confidence interval; CVD, cardiovascular disease; CKD, chronic kidney disease; MetS, metabolic syndrome.

The association between individual and cumulative CKM syndrome traits and incident MASLD hospitalization and other MALOs was also estimated (Figure 2). After multivariable adjustment, individuals with clinical CVD, stage 3a-5 CKD, or MetS were each separately associated with a heightened risk of incident MASLD hospitalization in comparison with no individual CKM syndrome trait, with HRs (95% CIs) of 1.89 (1.73, 2.06), 1.45 (1.24, 1.69), and 3.41 (3.22, 3.62), respectively, while the HRs of incident MASLD hospitalization were 3.31 (3.10, 3.52), 4.67 (4.20, 5.19), and 7.27 (5.46, 9.70), respectively, for those with 1, 2, and 3 CKM syndrome traits compared to those without. Additionally, analogous associations appeared in severe liver disease hospitalization and liver-specific mortality outcomes (Figure 2).

### 3.4 Mediation analyses of amino acids and free fatty acids with CKM-MALOs association

**Figure 3:**
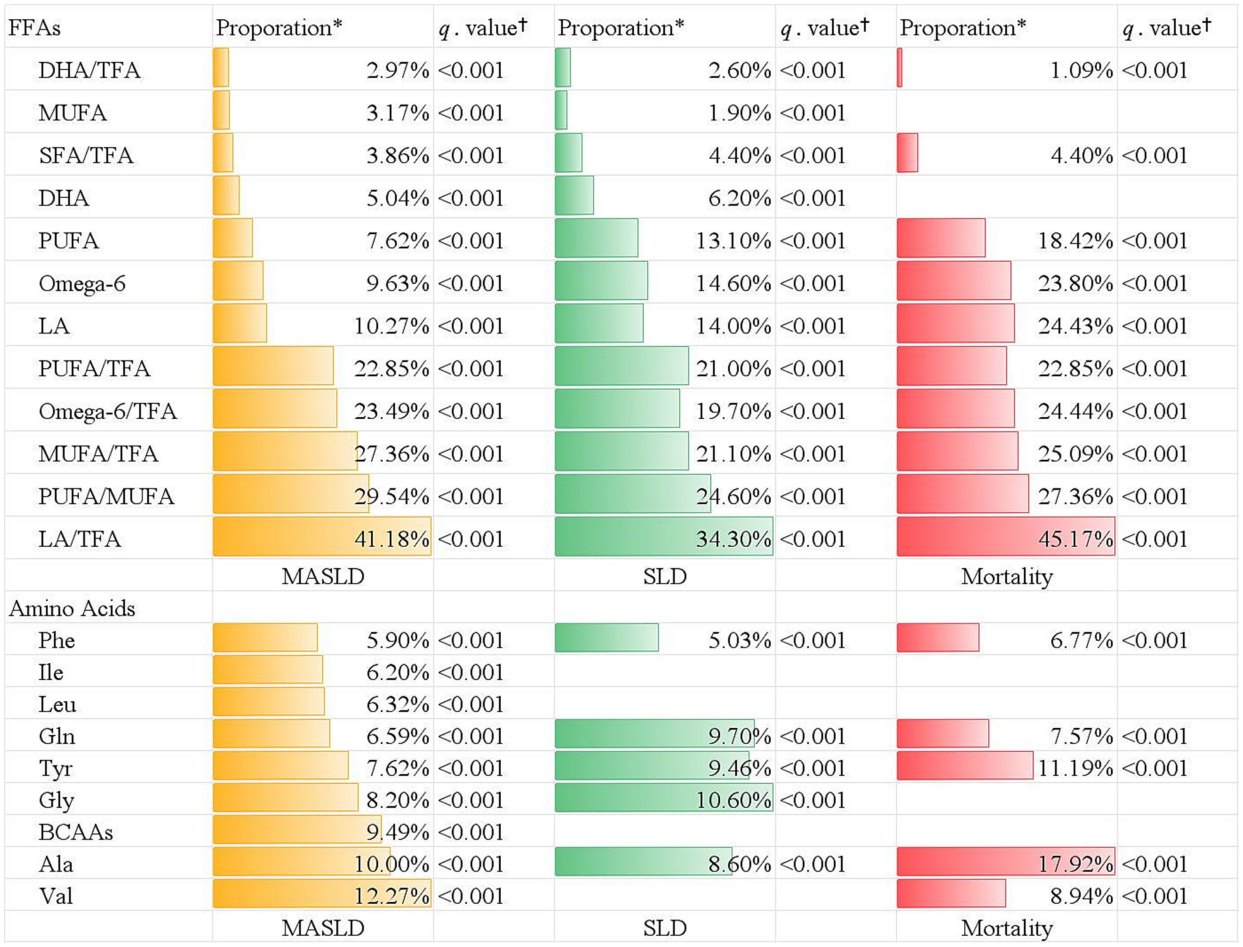
The mediation effect of plasma free fatty acids and amino acids on the association between CKM stage and different outcomes. *Indicates the proportion of mediation. ^†^indicates Benjamini–Hochberg adjusted *q-*value. TFA, total fatty acid; PUFA, polyunsaturated fatty acid; MUFA, monounsaturated fatty acid; SFA, saturated fatty acid; DHA, docosahexaenoic acid; omega-3, omega-3 fatty acid; omega-6, omega-6 fatty acid.

In the mediation analyses, there were 9 types of amino acids and 12 types of FFA biomarkers playing a mediating role in the CKM-MASLD association (Figure 3). The strongest amino acid mediator was valine (proportion of mediation [prop.] = 12.27%; *q* < 0.001), and the strongest fatty acid biomarker was LA/TFA (prop = 41.18%; *q* < 0.001). Similarly, a total of 5 amino acids and 12 FFAs biomarkers were identified as significant mediators in terms of CKM-SLD association (Figure 3). The ratios of LA/TFA, PUFA/MUFA, MUFA/TFA, PUFA/TFA, omega-6/TFA, and tyrosine accounted for 34.30, 24.60, 21.10, 21.00, 19.70, and 9.46% of the association between CKM stage and SLD, respectively (all *q* < 0.001).

Additionally, there were five types of amino acids mediators, in which alanine was the strongest mediator and accounted for 17.92% of the CKM-liver-specific mortality association (Figure 3). In 10 types of FFA mediators, the ratios of LA/TFA, PUFA/TFA, MUFA/TFA, omega-6/TFA, and LA explained 45.17, 27.36, 25.09, 24.44, and 24.43% of the association between CKM stage and liver-specific mortality, respectively (all *q* < 0.001) (Figure 3).

### 3.5 Subgroup and sensitivity analyses

As shown in Supplementary Figure S5, the association between CKM stage and incident MASLD hospitalization was modified by age and cumulative dietary risk (both *p*-interaction < 0.05). In addition, the association between CKM stage and severe liver disease hospitalization was significantly modified by age and drinking status (Supplementary Figure S6) (both *p*-interaction < 0.05). Of note, current drinkers exhibited a lower risk of severe liver disease hospitalization. Similarly, never and previous smokers had a higher risk of liver-specific mortality than current smokers (Supplementary Figure S7) (HR = 5.27, 95% CI:1.30, 21.38 versus HR = 2.75, 95% CI: 0.36, 20.28). These findings should be interpreted cautiously due to potential residual confounding and survivor bias. The findings of this study remained consistent across a series of sensitivity analyses (Supplementary Figures S7–S15).

## 4 Discussion

Several meaningful findings were observed in the current research. First, progressive CKM syndrome stage was associated with heightened risks of incident hospitalization for MASLD and severe liver disease, as well as an increased liver-specific mortality. After multiple adjustments for confounding factors, including sex, age, ethnicity, education level, TDI, sleep duration, physical activity intensity, smoking status, drinking status, and CDRFS, these associations were significant. Second, CKM syndrome traits were differentially and cumulatively associated with the risks of incident MASLD hospitalization and other MALOs. Third, amino acids and FFAs partially mediated the association between CKM and incident hospitalization for MASLD, severe liver disease, and liver-specific mortality, with tyrosine and alanine identified as the major amino acid mediators and the linoleic acid-to-total fatty acid percentage as the strongest fatty acid biomarker. Fourth, after sensitivity analyses, the associations between CKM stage and incident MALOs remained robust.

To the best of our knowledge, the present study is the first population-based prospective longitudinal research to quantify associations between different CKM syndrome stages and incident MASLD hospitalization, using a new staging structure rather than individual diseases in isolation. Existing studies have primarily demonstrated bidirectional relationships between individual diseases or risk factors (including diabetes, obesity, and CVD) and MASLD. On the one hand, obesity and diabetes are among the most significant drivers of MASLD development; approximately 65% of diabetes patients and up to 80% of obese individuals have MASLD (2, 8, 9). In addition, the existence of diabetes or CVD risk factors heightens MASLD risk (9, 28). A previous study demonstrated that Framingham Heart Study participants with certain baseline conditions had an elevated risk of developing fatty liver, including hypertension (OR = 3.34; 95% CI: 2.04, 5.49), MetS (OR = 4.63; 95% CI: 2.87, 7.47), IFG (OR = 2.92; 95% CI: 1.76, 4.82), and diabetes (OR = 4.15; 95% CI: 1.19, 14.46) (28), which is in line with our additional analyses. On the other hand, MASLD can increase the risks of diabetes, CVD, and CKD (29–32). Pooled data from 129 studies established that, compared to the general population, persons suffering from MASLD had increased rates of diabetes mellitus (HR = 2.56; 95% CI: 2.10, 3.13, *p* < 0.01), CKD (HR = 1.38, 95% CI: 1.27, 1.50, *p* < 0.01), and CVD (HR = 1.43, 95% CI: 1.27, 1.60, *p* < 0.01) (31). In addition, a current cross-sectional survey based on NHANES data indicated that persons with MASLD showed a heightened prevalence of CKM stage 2 and stage 3 (*p* < 0.001) compared to the general population; no significant difference was observed in those with CKM stage 4 (*p* = 0.12) (33). However, existing studies have either focused solely on the relationships between individual diseases and MASLD or failed to establish temporal associations between CKM stages and future liver outcomes. To address these gaps, we used the novel CKM staging system to capture integrated risk rather than individual diseases. With a larger sample size and prospective design, the current study focused on severe MASLD requiring hospitalization and expanded on this finding by demonstrating the longitudinal association between higher CKM stage and an increased risk of incident MASLD hospitalization.

Furthermore, this study is the first to reveal the positive associations between CKM stage and severe liver disease hospitalization and liver-specific mortality. Emerging evidence has shown that the presence of diabetes and other MetS traits is associated with elevated risks of liver cirrhosis and liver cancer (9, 34, 35). Additionally, persons with the coexistence of diabetes or other metabolic risk factors, such as CKD and CVD, have 2.27-fold and 1.95-fold higher risks of mortality, respectively, than persons with diabetes alone (35). Furthermore, another prospective study revealed that the incidence of adverse liver outcomes increases with a greater number of MetS traits (HR = 1.28, 95% CI: 1.23, 1.33) (36). In line with previous studies, we also found that CKM components were individually and cumulatively associated with the risks of severe liver disease and liver-specific mortality. However, those studies concentrated on individual or combined cardiometabolic risk factors, lacking a more comprehensive evaluation of CKM stage progression. Utilizing the CKM syndrome stage framework outlined in the Presidential Advisory from the American Heart Association, this study demonstrated a positive association between CKM progression and risks of severe liver disease and liver-specific mortality.

There are some biologically plausible mechanisms underlying the association between CKM syndrome and MASLD hospitalization and other major adverse liver outcomes. These may be partly due to overlapping pathophysiological mechanisms, including lipid toxicity, oxidative stress, insulin resistance, and inflammation (7, 8). CKM syndrome stems from excess or dysfunctional adipose tissue and obesity, which are among the most important factors in the natural history of MASLD (7, 8). The pro-inflammatory and pro-oxidative products secreted by dysfunctional adipose tissue can interfere with both extracellular and intracellular metabolism, particularly lipid metabolism (5). The resulting excess of free fatty acids inhibits liver insulin sensitivity and facilitates lipogenesis, causing steatosis and cirrhosis (37). This process further worsens inflammation, oxidative stress, and insulin resistance, which then damage the arterial, cardiac, and kidney tissues, ultimately resulting in the progression of CKM syndrome (7). Furthermore, the connections between these mechanisms are complex and multifaceted. For instance, infiltration of visceral adipose tissue by pro-inflammatory macrophages establishes a feed-forward cycle wherein adipose tissue inflammation exacerbates systemic insulin resistance (7, 38), while this metabolic dysfunction, particularly in insulin-sensitive organs (adipose tissue and pancreas), potentiates hepatic gluconeogenesis, subsequently fueling hepatic inflammatory responses and fibrotic progression (39). Finally, all these mechanisms bridge the association between CKM syndrome and MASLD and other liver outcomes.

Of note, this study revealed that a series of amino acids and FFAs partially mediated the associations between CKM stages and MALO risks. This finding suggests that these plasma metabolites may represent a critical biological pathway linking CKM syndrome to hepatic pathology. However, no consistent conclusion has been reached regarding the association between circulating metabolites and MASLD (12, 13, 40, 41). Interestingly, a previous study observed contradictory results in observational analysis and Mendelian randomization (MR) analysis, in which the observational analysis indicated a significant association with MASLD, while the MR analysis did not support a causal role for BCAAs or tyrosine on MASLD (13). This discrepancy could be explained by the fact that the observational study was confounded by factors such as insulin resistance, increased dietary protein intake, and protein catabolism (13, 40). Even so, the observed mediation effects are still biologically plausible due to multi-directional mechanisms. As noted, pathophysiologic processes such as lipotoxicity and insulin resistance—the core drivers of CKM syndrome (6, 7)—have been known to alter plasma metabolism (especially FFAs) (42) and facilitate MASLD pathogenesis (37, 42). Meanwhile, specific metabolites like FFAs can activate different pathways in various liver cells, aggravate lipotoxicity, and promote insulin resistance (37), forming a feedback cycle similar to that mentioned above.

The key strengths of our research include the application of updated CVD risk prediction equations, a prospective study design, a nationally representative sample of UK adults, a substantial sample size, prolonged follow-up, the assessment of an extensive set of potential confounding factors, and the examination of mediation by plasma metabolites. Nevertheless, several limitations should be noted. First, potential reverse causality might exist in the current study. However, a 2-year landmark analysis was used in the sensitivity analyses to investigate the robustness of the results. Second, this study focused on severe MASLD requiring hospitalization due to its marked heterogeneity in progression rates and outcomes, which could introduce potential selection bias. Further research involving all MASLD patients would help improve generalizability. Third, participants without CKM syndrome were excluded, which may affect the generalization of our conclusions to some extent. Fourth, this study could not explain how dynamic changes in the CKM syndrome stage affect MALO risk, despite the association between baseline CKM and MALO risk remaining significant over time (Supplementary Tables S13, S14). Further trajectory analyses incorporating dynamic information will help validate our results. Fifth, the inability to incorporate insulin resistance-related biomarkers as covariates-either because they were unavailable in the UK Biobank (e.g., homeostasis model assessment of insulin resistance) or were excluded to avoid multicollinearity (e.g., triglyceride-glucose index)-may introduce residual confounding. Sixth, 94.5% of participants in this cohort study were white, and our results may not be generalizable to other ethnic groups. Seventh, within subgroup analyses, never and previous smokers exhibited a higher liver-specific mortality risk than current smokers. Similarly, current drinkers showed a lower risk of severe liver disease. These findings should be interpreted with caution due to potential residual confounding (including dose–effect relationship, years of consumption, and reasons for quitting) and survivor bias, as high-risk current users may have already died from competing causes such as lung cancer.

## 5 Conclusion

This study provides longitudinal evidence on positive associations between CKM syndrome stage and MASLD hospitalization, as well as other major adverse liver outcomes. FFAs and amino acids partially mediate these associations. Given these findings, patients at advanced CKM stages may represent a high-risk group who could benefit from enhanced vigilance for liver disease, facilitating secondary prevention efforts.

## Data Availability

Publicly available datasets were analyzed in this study. This data can be found AT: http://www.ukbiobank.ac.uk/register-apply.

## Footnotes

^1^ https://www.ukbiobank.ac.uk

## Glossary

CKM syndrome: cardiovascular-kidney-metabolic syndrome
MASLD: metabolic dysfunction-associated fatty liver disease
HCC: hepatocellular carcinoma
CKD: chronic kidney disease
CVD: cardiovascular disease
MALOs: major adverse liver outcomes
FFAs: free fatty acids
BCAAs: branched-chain amino acids
NMR: nuclear magnetic resonance
MetS: metabolic syndrome
HbA1c: glycated hemoglobin
KDIGO: Kidney Disease Improving Global Outcomes
UKB: UK Biobank
ICD-10: International Statistical Classification of Diseases and Related Health Problems
10th revision
TDI: Townsend deprivation index
CRP: C-reactive protein
AST: aspartate aminotransferase
ALT: alanine aminotransferase
eGFR: estimated glomerular filtration rate
MASH: metabolic dysfunction-associated steatohepatitis
CDRFS: cumulative dietary risk factor score
NVQ: National Vocational Qualification
HND: Higher National Diploma
HNC: Higher National Certificates
TFA: total fatty acid
PUFA: polyunsaturated fatty acid
MUFA: monounsaturated fatty acid
SFA: saturated fatty acid
DHA: docosahexaenoic acid
Apo A: apolipoprotein A
Apo B: apolipoprotein B
SLD: severe liver disease
