# Supplementary material for: Cardiovascular-kidney-metabolic progression associated with major adverse liver outcomes: mediating roles of plasma metabolites
Source: Front Nutr. 2025 Oct 20;12:1675899. doi: 10.3389/fnut.2025.1675899 (PMC12580617; doi:10.3389/fnut.2025.1675899)
Supplement: Supplementary file 1 [file Table_1.DOCX]

**Supplementary Online Content**

Table S1. Definitions of CKM conditions

Table S2. Definitions of CKM syndrome stage.

Table S3. ICD-10 codes for diseases used as exclusion criteria.

Table S4. ICD-10 codes for outcomes of interest.

Table S5. Baseline characteristics of inclusion and exclusion participants

Table S6. Baseline characteristics of participants across main analyses and mediation analyses groups

Table S7. HRs for major adverse liver outcomes by CKM syndrome stage considering death as a competing risk

Table S8. HRs for major adverse liver outcomes by CKM syndrome stage using IPTW models

Table S9.Comparison of CKM Staging Using the AHA PREVENT and QRISK3 Risk Scores

Table S10.Agreement Between AHA PREVENT and QRISK3-Based CKM Staging

Table S11.HRs for major adverse liver outcomes by CKM syndrome stage using QRISK 3 score to predict the 10-year risk of cardiovascular

Table S12. HRs for study outcomes by CKM syndrome stage after excluding participants suffered from study outcomes or died within 2 years of follow-up.

Table S13. HRs for study outcomes by CKM syndrome stage after excluding participants suffered from study outcomes or died within 5 years of follow-up.

Table S14. HRs for study outcomes by CKM syndrome stage after excluding participants suffered from study outcomes or died within 7 years of follow-up.

Table S15. HRs for study outcomes by CKM syndrome stage after missing coviarates imputed using multiple imputation by chained equations

Table S1. Definitions of CKM conditions

| CKM  conditions | Definition | Indicators of each conditions | Definition for each indicators |
| --- | --- | --- | --- |
| CVD | Individuals with clinical CVD or  subclinical CVD | Clinical CVD | History of chronic heart failure, coronary heart disease, heart attack, atrial fibrillation, or stroke |
|  |  | Subclinical CVD | Any of the following criterion is met:  1) Very high-risk CKD in KDIGO classification: UACR ≥ 300 mg/g and eGFR ≤ 45-59 ml/min/1.73m^2^, UACR ≥ 30 mg/g and eGFR ≤ 30-44  ml/min/1.73m^2^, or eGFR ≤ 29 ml/min/1.73m^2^.  2) Predicted 10-year CVD risk ≥ 20% |
| Kidney  diseases | Individuals with CKD | CKD | Moderate-to-high-risk CKD in KDIGO classification: UACR ≥ 30 mg/g and eGFR ≥ 60 ml/min/1.73m^2^, UACR < 300 mg/g and eGFR ≤ 45-59 ml/min/1.73m^2^, or UACR < 30 mg/g and eGFR ≤ 30-44 ml/min/1.73m^2^. |
| Metabolic  disorders | Individuals with overweight / obesity, abdominal obesity, prediabetes, diabetes, hypertension, hypertriglyceridemia or MetS | Overweight/obesity | BMI ≥25 kg/m^2^ |
|  |  | Abdominal obesity | Waist circumference ≥88/102 cm in female/male |
|  |  | Prediabetes | Fasting blood glucose ≥ 100-124 mg/dL or HbA1c ≥ 5.7%-6.4% and without self-reported diagnosis of diabetes, or use of insulin |
|  |  | Diabetes | Fasting blood glucose ≥ 125 mg/dL or HbA1c ≥ 6.5% or self-reported diagnosis of diabetes, or use of insulin |
|  |  | Hypertension | SBP ≥130 mm Hg or DBP ≥80 mm Hg or self-reported diagnosis of hypertension or use of antihypertensive medications |
|  |  | Hypertriglyceridemia | Triglycerides ≥ 135 mg/dL |
|  |  | MetS | MetS is defined by the presence of 3 or more of the following:  1) Waist circumference ≥88/102 cm in female/male.  2) HDL cholesterol < 50/40 mg/dL in female/male.  3) Triglycerides ≥150 mg/dL.  4) Elevated blood pressure (SBP ≥130 mm Hg or DBP ≥80 mmHg and/or use of antihypertensive medications)  5) Fasting blood glucose ≥100 mg/dL |

Abbreviations: CKM: cardiovascular-kidney-metabolic; CVD: cardiovascular disease; CKD: chronic kidney disease; KDIGO: The Kidney Disease: Improving Global Outcomes; UACR: urinary albumin to creatinine ratio; eGFR: estimated glomerular filtration rate; DBP: diastolic blood pressure; BMI: body mass index;HbA1c:glycosylated hemoglobin, type A1C;HDL: high-density lipoprotein; MetS: metabolic syndrome; SBP: systolic blood pressure;

Table S2. Definitions of CKM syndrome stage.

| CKM stages | CKM stages | CKM stages | CKM stages |
| --- | --- | --- | --- |
| Stage 0: No  CKM risk factors | Individuals with normal BMI and waist circumference, normoglycemia,  normotension, a normal lipid profile,  and no evidence of CKD or subclinical  or clinical CVD | All criteria are met | BMI <25 kg/m2 |
|  |  |  | Waist circumference <88/102 cm in female/male |
|  |  |  | Fasting blood glucose < 100 mg/dL and HbA1c < 5.7% and without self-reported diagnosis of diabetes, use of insulin |
|  |  |  | SBP <130 mm Hg and DBP <80 mm Hg without self-reported diagnosis of hypertension or use of antihypertensive medications |
|  |  |  | HDL cholesterol >50/40 mg/dL in female/male and triglycerides < 150 mg/dL |
|  |  |  | Low-risk CKD in KDIGO classification according to eGFR and  UACR: UACR < 30 mg/g and eGFR ≥ 60 ml/min/1.73m2. |
|  |  |  | Predicted 10-year CVD risk < 20% |
|  |  |  | No clinical CVD |
| Stage 1: Excess  or dysfunctional  adiposity | Individuals with overweight/obesity,  abdominal obesity, or dysfunctional  adipose tissue, without the presence of  other metabolic risk factors or CKD | Any of the three criteria is met | Overweight/obesity |
|  |  |  | Abdominal obesity |
|  |  |  | Prediabetes |
|  |  | All criteria are met | SBP <130 mm Hg and DBP <80 mm Hg without self-reported  diagnosis of hypertension or use of antihypertensive medications |
|  |  |  | HDL cholesterol >50/40 mg/dL in female/male and triglycerides < 150 mg/dL |
|  |  |  | Low-risk CKD in KDIGO classification according to eGFR and  UACR: UACR < 30 mg/g and eGFR ≥ 60 ml/min/1.73m2. |
|  |  |  | Predicted 10-year CVD risk < 20% |
|  |  |  | No clinical CVD |
| Stage 2:  Metabolic risk  factors and CKD | Individuals with metabolic risk factors (hypertriglyceridemia, hypertension, MetS, diabetes), or CKD | Any of the five criteria is met | Hypertriglyceridemia |
|  |  |  | Hypertension |
|  |  |  | diabetes |
|  |  |  | MetS |
|  |  |  | Moderate-to-high-risk CKD in KDIGO classification |
|  |  | All criteria are met | No very high-risk CKD in KDIGO classification |
|  |  |  | Predicted 10-year CVD risk < 20% |
|  |  |  | No clinical CVD |
| Stage 3:  Subclinical CVD  in CKM | Subclinical CVD among individuals  with excess/dysfunctional adiposity,  other metabolic risk factors, or CKD | Any of the two criteria is met | Very high-risk CKD in KDIGO classification |
|  |  |  | Predicted 10-year CVD risk ≥ 20% |
|  |  | All criteria are met | Overweight/obesity |
|  |  |  | Abdominal obesity |
|  |  |  | Prediabetes |
|  |  |  | Hypertriglyceridemia |
|  |  |  | Hypertension |
|  |  |  | diabetes |
|  |  |  | MetS |
|  |  |  | Moderate-to-high-risk CKD in KDIGO classification |
|  |  | The criterion is met | No clinical CVD |
| Stage 4: Clinical  CVD in CKM | Clinical CVD among individuals with excess/dysfunctional adiposity,  other metabolic risk factors, or CKD | The criterion is met | Clinical CVD |
|  |  | Any of the nine criteria is met | Overweight/obesity |
|  |  |  | Abdominal obesity |
|  |  |  | Prediabetes |
|  |  |  | Hypertriglyceridemia |
|  |  |  | Hypertension |
|  |  |  | diabetes |
|  |  |  | MetS |
|  |  |  | Moderate-to-high-risk CKD in KDIGO classification |
|  |  |  | Very high-risk CKD in KDIGO classification |

Abbreviation: CKM: cardiovascular-kidney-metabolic; CVD: cardiovascular disease; BMI: body mass index; HbA1c:glycosylated hemoglobin, type A1C; SBP: systolic blood pressure;

HDL: high-density lipoprotein; CKD: chronic kidney disease; KDIGO: The Kidney Disease: Improving Global Outcomes; UACR: urinary albumin to creatinine ratio; eGFR: estimated glomerular filtration rate; DBP: diastolic blood pressure;MetS: metabolic syndrome;

Table S3. ICD-10 codes for diseases used as exclusion criteria.

| **Prevalent chronic liver diseases** | **ICD-10 code** |
| --- | --- |
| Prevalent MASLD^[1]^ | K75.8, K76.0 |
| Prevalent cirrhosis | K70.2, K70.3, K70.4, K74.0, K74.1, K74.2, K74.6, K76.6, and I85.0, I85.9 |
| Prevalent HCC, and other liver cancer | C22.0, C22.1, C22.2, C22.3, C22.4, C22.7, C22.9 |
| **Alcohol/drug use disorder** |  |
| Codes associated with alcohol use disorder | F10 |
| Codes associated with somatic consequences of alcohol (except alcoholic liver disease) | E24.4, G62.1, I42.6, K29.2, G31.2, G72.1, K85.2, K86.0, T51.0, T51.9, Y57.3, X65, Z50.2, Z71.4, Z72.1 |
| Codes associated with drug use disorders except nicotine/caffeine | F11, F12, F13, F14, F16, F18, F19 |
| **Other chronic liver diseases** |  |
| Alcoholic liver disease | K70 |
| Viral hepatitis | B15, B16, B17, B18, B19 |
| Autoimmune liver disease (autoimmune hepatitis,primary biliary cholangitis, primary sclerosing cholangitis) | K83.0, K74.3, K75.4 |
| Hemochromatosis | E83.1 |
| Wilson | E83.0 |
| Alpha-1-antitrypsin deficiency | E88.0 |
| Budd-Chiari | I82.0, K76.5 |
| Chronic hepatitis, unspecified | K73.9, K73.2 |
| Secondary or unspecified biliary cirrhosis | K74.4, K74.5 |
| Liver transplantation | Z94.4 |

Reference

1. Hagström H, Adams LA, Allen AM, Byrne CD, Chang Y, Grønbaek H, et al. Administrative Coding in Electronic Health Care Record-Based Research of NAFLD: An Expert Panel Consensus Statement. Hepatol Baltim Md. 2021;74:474–82.

Table S4. ICD-10 codes for outcomes of interest^[1]^

| Outcomes of interest | UK Biobank Data-Field | ICD-10 code |
| --- | --- | --- |
| MASLD | Diagnoses - ICD10: 41270 | K75.8, K76.0 |
| Severe liver disease |  |  |
| Cirrhosis | Diagnoses - ICD10: 41270 | K74.0, K74.1, K74.2, K74.6, K70.2, K70.3, K70.4, K76.6, and I85 |
| Hepatocellular carcinoma | Diagnoses - ICD10: 41270 | C22.0 |
| Intrahepatic cholangiocarcinoma | Diagnoses - ICD10: 41270 | C22.1 |
| Other liver cancer | Diagnoses - ICD10: 41270 | C22.2-C22.4, C22.7, C22.9 |
| Liver-specific mortality | Underlying (primary) cause of death: 40001  Contributory (secondary) causes of death: 40002 | K74.0, K74.1, K74.2, K74.6, K70.2, K70.3, K70.4, K75.8, K76.0, K76.6, I85, C22.0, C22.1, C22.2-C22.4, C22.7, C22.9 |

Table S5. Baseline characteristics of inclusion and exclusion participants

| **Characteristic** | **Group** | | |
| --- | --- | --- | --- |
|  | **Total** | **Inclusion** | **Exclusion** |
| Participants | 502364 | 415713 | 86651 |
| CKM stage |  |  |  |
| Stage 0 | 5269 (1.0) | 5172 (1.2) | 97 (0.1) |
| Stage 1 | 4413 (0.9) | 4313 (1.0) | 100 (0.1) |
| Stage 2 | 349123 (69.5) | 340528 (81.9) | 8595 (9.9) |
| Stage 3 | 108317 (21.6) | 38505 (9.3) | 69812 (80.6) |
| Stage 4 | 33491 (6.7) | 27195 (6.5) | 6296 (7.3) |
| Missing | 1751 (0.3) | 0 (0.0) | 1751 (2.0) |
| Sex (%) |  |  |  |
| Female | 273297 (54.4) | 223781 (53.8) | 49516 (57.1) |
| Male | 229066 (45.6) | 191932 (46.2) | 37134 (42.9) |
| Missing | 1 (0.0) | 0 (0.0) | 1 (0.0) |
| Age, mean (SD) | 56.53 (8.09) | 56.56 (8.08) | 56.41 (8.14) |
| Townsend Deprivation Index, mean (SD) | -1.29 (3.09) | -1.34 (3.07) | -1.08 (3.21) |
| Ethnicity (%) |  |  |  |
| White | 472569 (94.1) | 392741 (94.5) | 79828 (92.1) |
| Non-white | 27018 (5.4) | 21565 (5.2) | 5453 (6.3) |
| Missing | 2777 (0.6) | 1407 (0.3) | 1370 (1.6) |
| Education (%) |  |  |  |
| College or university degree | 161102 (32.1) | 134483 (32.3) | 26619 (30.7) |
| Other degree | 331132 (65.9) | 276912 (66.6) | 54220 (62.6) |
| Missing | 10130 (2.0) | 4318 (1.0) | 5812 (6.7) |
| Sleep duration, n (%) |  |  |  |
| N<7h | 123214 (24.5) | 101768 (24.5) | 21446 (24.7) |
| 7h>=N>=9h | 365694 (72.8) | 304117 (73.2) | 61577 (71.1) |
| N>9h | 9241 (1.8) | 7359 (1.8) | 1882 (2.2) |
| Missing | 4215 (0.8) | 2469 (0.6) | 1746 (2.0) |
| Physical activity, n (%) |  |  |  |
| Low | 76190 (15.2) | 63056 (15.2) | 13134 (15.2) |
| Moderate | 163986 (32.6) | 137269 (33.0) | 26717 (30.8) |
| High | 162095 (32.3) | 136416 (32.8) | 25679 (29.6) |
| Missing | 100093 (19.9) | 78972 (19.0) | 21121 (24.4) |
| Smoking status, n (%) |  |  |  |
| Never | 273449 (54.4) | 227733 (54.8) | 45716 (52.8) |
| Previous | 173006 (34.4) | 144384 (34.7) | 28622 (33.0) |
| Current | 52960 (10.5) | 43596 (10.5) | 9364 (10.8) |
| Missing | 2949 (0.6) | 0 (0.0) | 2949 (3.4) |
| Drinking status, n (%) |  |  |  |
| Never | 22378 (4.5) | 18023 (4.3) | 4355 (5.0) |
| Previous | 18093 (3.6) | 14270 (3.4) | 3823 (4.4) |
| Current | 460240 (91.6) | 382977 (92.1) | 77263 (89.2) |
| Missing | 1653(0.3) | 443 (0.1) | 1210(1.4) |
| Diet score,(mean (SD)) | 5.08 (1.48) | 5.08 (1.49) | 5.06 (1.44) |

Table S6. Baseline characteristics of participants across main analyses and mediation analyses groups

| **Characteristic** | **Group** | | |
| --- | --- | --- | --- |
|  | **Total** | **Main analyses** | **Mediation analyses** |
| Participants | 646795 | 415713 | 231082 |
| CKM stage |  |  |  |
| Stage 0 | 7980 (1.2) | 5172 (1.2) | 2808 (1.2) |
| Stage 1 | 6696 (1.0) | 4313 (1.0) | 2383 (1.0) |
| Stage 2 | 529759 (81.9) | 340528 (81.9) | 189231 (81.9) |
| Stage 3 | 59679 (9.2) | 38505 (9.3) | 21174 (9.2) |
| Stage 4 | 42681 (6.6) | 27195 (6.5) | 15486 (6.7) |
| Male (%) | 299152 (46.3) | 191932 (46.2) | 107220 (46.4) |
| Age, mean (SD) | 56.56 (8.08) | 56.56 (8.08) | 56.57 (8.08) |
| Townsend Deprivation Index, mean (SD) | -1.36 (3.06) | -1.34 (3.07) | -1.39 (3.05) |
| Ethnicity (%) |  |  |  |
| White | 612024 (94.6) | 392741 (94.5) | 219283 (94.9) |
| Non-white | 32583 (5.0) | 21565 (5.2) | 11018 (4.8) |
| Missing | 2188 (0.3) | 1407 (0.3) | 781 (0.3) |
| Education (%) |  |  |  |
| College or university degree | 207553 (32.1) | 134483 (32.3) | 73070 (31.6) |
| Other degree | 432523 (66.9) | 276912 (66.6) | 155611 (67.3) |
| Missing | 6719 (1.0) | 4318 (1.0) | 2401 (1.0) |
| Sleep duration, n (%) |  |  |  |
| N<7h | 158359 (24.5) | 101768 (24.5) | 56591 (24.5) |
| 7h>=N>=9h | 473108 (73.1) | 304117 (73.2) | 168991 (73.1) |
| N>9h | 11491 (1.8) | 7359 (1.8) | 4132 (1.8) |
| Missing | 3837 (0.6) | 2469 (0.6) | 1368 (0.6) |
| Physical activity, n (%) |  |  |  |
| Low | 98222 (15.2) | 63056 (15.2) | 35166 (15.2) |
| Moderate | 212810 (32.9) | 137269 (33.0) | 75541 (32.7) |
| High | 212454 (32.8) | 136416 (32.8) | 76038 (32.9) |
| Missing | 123309 (19.1) | 78972 (19.0) | 44337 (19.2) |
| Smoking status, n (%) |  |  |  |
| Never | 354122 (54.8) | 227733 (54.8) | 126389 (54.7) |
| Previous | 224833 (34.8) | 144384 (34.7) | 80449 (34.8) |
| Current | 67840 (10.5) | 43596 (10.5) | 24244 (10.5) |
| Drinking status, n (%) |  |  |  |
| Never | 27847 (4.3) | 18023 (4.3) | 9824 (4.3) |
| Previous | 22126 (3.4) | 14270 (3.4) | 7856 (3.4) |
| Current | 596118 (92.2) | 382977 (92.1) | 213141 (92.2) |
| Missing | 704 (0.1) | 443 (0.1) | 261 (0.1) |
| Diet score,(mean (SD)) | 5.08 (1.49) | 5.08 (1.49) | 5.08 (1.49) |

Continuous variables are reported as their mean (SD), while categorical variables are reported as their number (percentage). Higher educated indicates college, university, national vocational qualification, higher national diploma, higher national certificates or equivalent; CDRFS, cumulative dietary risk factor score; AST, aspartate aminotransferase; ALT, alanine aminotransferase;

Table S7. HRs for major adverse liver outcomes by CKM syndrome stage considering death as a competing risk

| **Model** | **CKM syndrome stage** | | | | |  |
| --- | --- | --- | --- | --- | --- | --- |
|  | **Stage 0** | **Stage 1** | **Stage 2** | **Stage 3** | **Stage 4** |  |
| MASLD |  |  |  |  |  |  |
| Events/Subjects | 14/ 5,172 | 34/4,313 | 3,448/340,528 | 691/38,505 | 658/27,195 |  |
| Model 2 | Reference | 2.65 (1.42-4.93) | 3.57 (2.11-6.03) | 5.78 (3.39-9.84) | 6.99 (4.10-11.91) |  |
| *p.* value | Reference | 0.002 | < 0.001 | < 0.001 | < 0.001 |  |
|  |  |  |  |  |  |  |
| Severe liver disease |  |  |  |  |  |  |
| Events/Subjects | 12/ 5,172 | 23/4,313 | 1,647/340,528 | 515/38,505 | 429/27,195 |  |
| Model 2 | Reference | 2.45 (1.02-5.88) | 1.62 (0.78-3.35) | 3.41 (1.64-7.10) | 3.63 (1.73-7.61) |  |
| *p.* value | Reference | 0.044 | 0.193 | 0.001 | < 0.001 |  |
|  |  |  |  |  |  |  |
| Liver-specific mortality* |  |  |  |  |  |  |
| Events/Subjects | 3/ 5,172 | 4/4,313 | 578/340,528 | 206/38,505 | 161/27,195 |  |
| Model 2 | Reference | 1.33(0.30–5.93) | 2.16(0.69–6.71) | 4.10(1.31–12.86) | 4.12(1.31–12.95) |  |
| *p.* value | Reference | 0.71 | 0.180 | 0.016 | 0.015 |  |

* Non-liver-related mortality was considered a competing risk for liver-related mortality.

Table S8. HRs for major adverse liver outcomes by CKM syndrome stage using IPTW models

| **Model** | **CKM syndrome stage** | | | | |  |
| --- | --- | --- | --- | --- | --- | --- |
|  | **Stage 0** | **Stage 1** | **Stage 2** | **Stage 3** | **Stage 4** |  |
| MASLD |  |  |  |  |  |  |
| Events/Subjects | 14/ 5,172 | 34/4,313 | 3448/340,528 | 691/38,505 | 658/27,195 |  |
| Model 2 | Reference | 1.66 (0.72-3.81) | 2.08 (1.00-4.35) | 3.29 (1.58-6.84) | 4.63 (2.21-9.69) |  |
| *p.* value | Reference | 0.235 | 0.050 | 0.001 | < 0.001 |  |
|  |  |  |  |  |  |  |
| Severe liver disease |  |  |  |  |  |  |
| Events/Subjects | 12/ 5,172 | 23/4,313 | 1647/340,528 | 515/38,505 | 429/27,195 |  |
| Model 2 | Reference | 2.45 (1.02-5.88) | 1.62 (0.78-3.35) | 3.41 (1.64-7.10) | 3.63 (1.73-7.61) |  |
| *p.* value | Reference | 0.044 | 0.193 | 0.001 | < 0.001 |  |
|  |  |  |  |  |  |  |
| Liver-specific mortality |  |  |  |  |  |  |
| Events/Subjects | 3/ 5,172 | 4/4,313 | 578/340,528 | 206/38,505 | 161/27,195 |  |
| Model 2 | Reference | 2.78 (0.54-14.30) | 3.57 (1.09-11.65) | 7.42 (2.23-24.65) | 6.49 (1.95-21.60) |  |
| *p.* value | Reference | 0.220 | 0.035 | 0.001 | 0.002 |  |

Table S9.Comparison of CKM Staging Using the AHA PREVENT and QRISK3 Risk Scores

|  | CKMs Stages Based on QRISK3 Risk Scores | | | | | | |
| --- | --- | --- | --- | --- | --- | --- | --- |
| CKM Stages Based on AHA PREVENT | Stage | 0 | 1 | 2 | 3 | 4 | Total |
|  | 0 | 5,144 | 0 | 0 | 2 | 0 | 5,146 |
|  | 1 | 0 | 4,264 | 0 | 49 | 0 | 4,313 |
|  | 2 | 0 | 0 | 310,126 | 30,402 | 0 | 340,528 |
|  | 3 | 6 | 87 | 17,863 | 20,517 | 0 | 38,473 |
|  | 4 | 0 | 0 | 0 | 0 | 27,195 | 27,195 |
| Total | | 5,150 | 4,351 | 327,989 | 50,970 | 27,195 | 415,655 |

Excluding 58 participants missing complete information for assessing QRISK3 Risk Scores

Table S10.Agreement Between AHA PREVENT and QRISK3-Based CKM Staging

| Agreement | Expected agreement | Kappa | Std. err. Z | Z | Prob>Z |
| --- | --- | --- | --- | --- | --- |
| 88.35% | 66.24% | 0.6551 | 0.0011 | 590.42 | <0.001 |

| **Model** | **CKM syndrome stage** | | | | | ***p* for trend** |
| --- | --- | --- | --- | --- | --- | --- |
|  | **Stage 0** | **Stage 1** | **Stage 2** | **Stage 3** | **Stage 4** |  |
| MASLD |  |  |  |  |  |  |
| Events/Subjects | 14/5,150 | 33 / 4,351 | 3,272 / 327,989 | 868/ 50,970 | 658/ 27,195 |  |
| model 1 | Reference | 2.68 (1.43-5.00) | 3.66 (2.17-6.20) | 6.33 (3.72-10.77) | 8.91 (5.24-15.17) | <0.001 |
| model 2 | Reference | 2.56 (1.37-4.79) | 3.56 (2.11-6.02) | 5.70 (3.35-9.70) | 7.77 (4.56-13.22) | <0.001 |
|  |  |  |  |  |  |  |
| Severe liver disease |  |  |  |  |  |  |
| Events/Subjects | 12/5,150 | 21/ 4,351 | 1,580/ 327,989 | 582/ 50,970 | 429 / 27,195 |  |
| Model 1 | Reference | 1.82 (0.89-3.70) | 1.70 (0.97-3.01) | 2.32 (1.30-4.13) | 3.68（2.06，6.55） | <0.001 |
| model 2 | Reference | 1.77 (0.87-3.59) | 1.70 (0.96-3.01) | 2.00 (1.13-3.57) | 3.22 (1.81-5.74) | <0.001 |
|  |  |  |  |  |  |  |
| Liver-specific mortality |  |  |  |  |  |  |
| Events/Subjects | 3 /5,150 | 4/ 4,351 | 559 / 327,989 | 225 / 50,970 | 161/ 27,195 |  |
| model 1 | Reference | 1.34 (0.30-6.01) | 2.25 (0.72-7.01) | 2.79 (0.89-8.77) | 4.48 (1.42-14.10) | <0.001 |
| model 2 | Reference | 1.29 (0.29-5.77) | 2.26 (0.73-7.04) | 2.33 (0.74-7.32) | 3.87 (1.23-12.19) | <0.001 |

Table S11.HRs for major adverse liver outcomes by CKM syndrome stage using QRISK 3 score to predict the 10-year cardiovascular risk

Table S12. HRs for study outcomes by CKM syndrome stage after excluding participants suffered from study outcomes or died within 2 years of follow-up.

| **Model** | **CKM syndrome stage** | | | | | ***p* for trend** |
| --- | --- | --- | --- | --- | --- | --- |
|  | **Stage 0** | **Stage 1** | **Stage 2** | **Stage 3** | **Stage 4** |  |
| MASLD |  |  |  |  |  |  |
| Events/Subjects | 14 / 5,157 | 33 / 4,291 | 3,298 / 339,187 | 649 / 38,117 | 624 / 26,751 |  |
| model 1 | Reference | 2.68 (1.43-5.01) | 3.47 (2.05-5.87) | 6.07 (3.57-10.33) | 7.94 (4.67-13.52) | <0.001 |
| model 2 | Reference | 2.57 (1.38-4.80) | 3.39 (2.01-5.74) | 5.53 (3.25-9.41) | 7.05 (4.14-12.01) | <0.001 |
|  |  |  |  |  |  |  |
| Severe liver disease |  |  |  |  |  |  |
| Events/Subjects | 10 / 5,157 | 21 / 4,291 | 1,538 / 339,187 | 485 / 38,117 | 398 / 26,751 |  |
| Model 1 | Reference | 2.21 (1.04-4.70) | 1.83 (0.98-3.41) | 3.91 (2.08-7.33) | 4.21 (2.24-7.91) | <0.001 |
| model 2 | Reference | 2.14 (1.01-4.55) | 1.82 (0.98-3.40) | 3.60 (1.92-6.75) | 3.85 (2.05-7.23) | <0.001 |
|  |  |  |  |  |  |  |
| Liver-specific mortality |  |  |  |  |  |  |
| Events/Subjects | 3 / 5,157 | 3 / 4,291 | 532/ 339,187 | 195 / 38,117 | 149/ 26,751 |  |
| model 1 | Reference | 1.03 (0.21-5.09) | 1.96(0.63-6.09) | 4.35(1.39-13.67) | 4.42 (1.40-13.92) | <0.001 |
| model 2 | Reference | 0.98 (0.20-4.85) | 1.95 (0.63-6.07) | 3.89 (1.24-12.21) | 4.03 (1.28-12.70) | <0.001 |

Abbreviation: CKM: cardiovascular-kidney-metabolic; MASLD, metabolic dysfunction-associated fatty liver disease;

Table S13. HRs for study outcomes by CKM syndrome stage after excluding participants suffered from study outcomes or died within 5 years of follow-up.

| **Model** | **CKM syndrome stage** | | | | | ***p* for trend** |
| --- | --- | --- | --- | --- | --- | --- |
|  | **Stage 0** | **Stage 1** | **Stage 2** | **Stage 3** | **Stage 4** |  |
| MASLD |  |  |  |  |  |  |
| Events/Subjects | 13 / 5,113 | 31 / 4,247 | 2,915 / 335,595 | 536 / 37,152 | 523 / 25,801 |  |
| model 1 | Reference | 2.72 (1.42-5.20) | 3.33 (1.93-5.75) | 5.53 (3.18-9.60) | 7.39 (4.25-12.84) | <0.001 |
| model 2 | Reference | 2.61 (1.37-5.00) | 3.26 (1.89-5.63) | 5.04 (2.90-8.76) | 6.60 (3.80-11.46) | <0.001 |
|  |  |  |  |  |  |  |
| Severe liver disease |  |  |  |  |  |  |
| Events/Subjects | 9 / 5,113 | 15 / 4,247 | 1,303 / 335,595 | 388 / 37,152 | 334 / 25,801 |  |
| Model 1 | Reference | 1.76 (0.77-4.01) | 1.72 (0.89-3.32) | 3.53 (1.82-6.86) | 4.00 (2.06-7.78) | <0.001 |
| model 2 | Reference | 1.70 (0.74-3.89) | 1.72 (0.89-3.32) | 3.26 (1.68-6.34) | 3.69 (1.90-7.18) | <0.001 |
|  |  |  |  |  |  |  |
| Liver-specific mortality |  |  |  |  |  |  |
| Events/Subjects | 3 / 5,113 | 2 / 4,247 | 436/ 335,595 | 152/ 37,152 | 123/ 25,801 |  |
| model 1 | Reference | 0.68 (0.11-4.09) | 1.60 (0.51-4.98) | 3.46 (1.10-10.90) | 3.70 (1.17-11.68) | <0.001 |
| model 2 | Reference | 0.65 (0.11-3.91) | 1.60 (0.51-4.99) | 3.10 (0.98-9.78) | 3.40 (1.08-10.76) | <0.001 |

Table S14. HRs for study outcomes by CKM syndrome stage after excluding participants suffered from study outcomes or died within 7 years of follow-up.

| **Model** | **CKM syndrome stage** | | | | | ***p* for trend** |
| --- | --- | --- | --- | --- | --- | --- |
|  | **Stage 0** | **Stage 1** | **Stage 2** | **Stage 3** | **Stage 4** |  |
| MASLD |  |  |  |  |  |  |
| Events/Subjects | 12 / 5,081 | 29 / 4,219 | 2,476 / 332,330 | 454 / 36,303 | 424 / 24,934 |  |
| model 1 | Reference | 2.77 (1.41-5.43) | 3.09 (1.75-5.45) | 5.17 (2.91-9.18) | 6.66 (3.74-11.84) | <0.001 |
| model 2 | Reference | 2.66 (1.36-5.22) | 3.03 (1.72-5.34) | 4.73 (2.66-8.41) | 5.96 (3.35-10.61) | <0.001 |
|  |  |  |  |  |  |  |
| Severe liver disease |  |  |  |  |  |  |
| Events/Subjects | 6 / 5,081 | 13 / 4,219 | 1,075 / 332,330 | 310 / 36,303 | 249 / 24,934 |  |
| Model 1 | Reference | 2.28 (0.87-6.01) | 2.14 (0.96-4.78) | 4.29 (1.91-9.65) | 4.54 (2.01-10.23) | <0.001 |
| model 2 | Reference | 2.22 (0.84-5.84) | 2.14 (0.96-4.78) | 3.95 (1.75-8.88) | 4.17 (1.85-9.40) | <0.001 |
|  |  |  |  |  |  |  |
| Liver-specific mortality |  |  |  |  |  |  |
| Events/Subjects | 2 / 5,081 | 2 / 4,219 | 351 / 332,330 | 114 / 36,303 | 87 / 24,934 |  |
| model 1 | Reference | 1.03 (0.14-7.30) | 1.92 (0.48-7.73) | 3.87 (0.95-15.74) | 3.89 (0.95-15.89) | <0.001 |
| model 2 | Reference | 0.98 (0.14-6.96) | 1.92 (0.48-7.72) | 3.46 (0.85-14.09) | 3.58 (0.88-14.62) | <0.001 |

Table S15. HRs for study outcomes by CKM syndrome stage after missing coviarates imputed using multiple imputation by chained equations

| **Model** | **CKM syndrome stage** | | | | | ***p* for trend** |
| --- | --- | --- | --- | --- | --- | --- |
|  | **Stage 0** | **Stage 1** | **Stage 2** | **Stage 3** | **Stage 4** |  |
| MASLD |  |  |  |  |  |  |
| Events/Subjects | 14/ 5,172 | 34/4,313 | 3448/340,528 | 691/38,505 | 658/27,195 |  |
| model 1 | Reference | 2.82 (1.52-5.26) | 3.72 (2.20-6.28) | 6.65 (3.91-11.31) | 8.64 (5.08-14.69) | <0.001 |
| model 2 | Reference | 2.70 (1.45-5.03) | 3.61 (2.14-6.11) | 6.02 (3.54-10.23) | 7.59 (4.46-12.91) | <0.001 |
|  |  |  |  |  |  |  |
| Severe liver disease |  |  |  |  |  |  |
| Events/Subjects | 12/ 5,172 | 23/4,313 | 1647/340,528 | 515/38,505 | 429/27,195 |  |
| Model 1 | Reference | 2.06 (1.02-4.14) | 1.67 (0.95-2.95) | 3.56 (2.00-6.33) | 3.89 (2.19-6.93) | <0.001 |
| model 2 | Reference | 1.98 (0.99-3.98) | 1.65 (0.94-2.92) | 3.26 (1.84-5.80) | 3.53 (1.98-6.29) | <0.001 |
|  |  |  |  |  |  |  |
| Liver-specific mortality |  |  |  |  |  |  |
| Events/Subjects | 3/ 5,172 | 4/4,313 | 578/340,528 | 206/38,505 | 161/27,195 |  |
| model 1 | Reference | 1.40 (0.31-6.27) | 2.19 (0.70-6.80) | 4.78 (1.52-15.01) | 4.97 (1.58-15.63) | <0.001 |
| model 2 | Reference | 1.33 (0.30-5.96) | 2.17 (0.70-6.74) | 4.22 (1.35-13.26) | 4.46 (1.42-14.03) | <0.001 |
