# Supplementary material for: Cardiovascular-kidney-metabolic progression associated with major adverse liver outcomes: mediating roles of plasma metabolites
Source: Front Nutr. 2025 Oct 20;12:1675899. doi: 10.3389/fnut.2025.1675899 (PMC12580617; doi:10.3389/fnut.2025.1675899)
Supplement: Supplementary file 2 [file Table_2.DOCX]

**Supplementary Online Content**

Figure S1.Directed acyclic graph

Figure S2 Ten year cumulative incidence of MASLD by CKM stages

Figure S3 Ten year cumulative incidence of severe liver disease by CKM stages

Figure S4 Ten year cumulative incidence of liver-related death by CKM stages

Figure S5. Stratified analyses of the associations of CKM stage with risk of MASLD

Figure S6. Stratified analyses of the associations of CKM stage with risk of severe liver disease

Figure S7. Stratified analyses of the associations of CKM stage with risk of liver-specific mortality


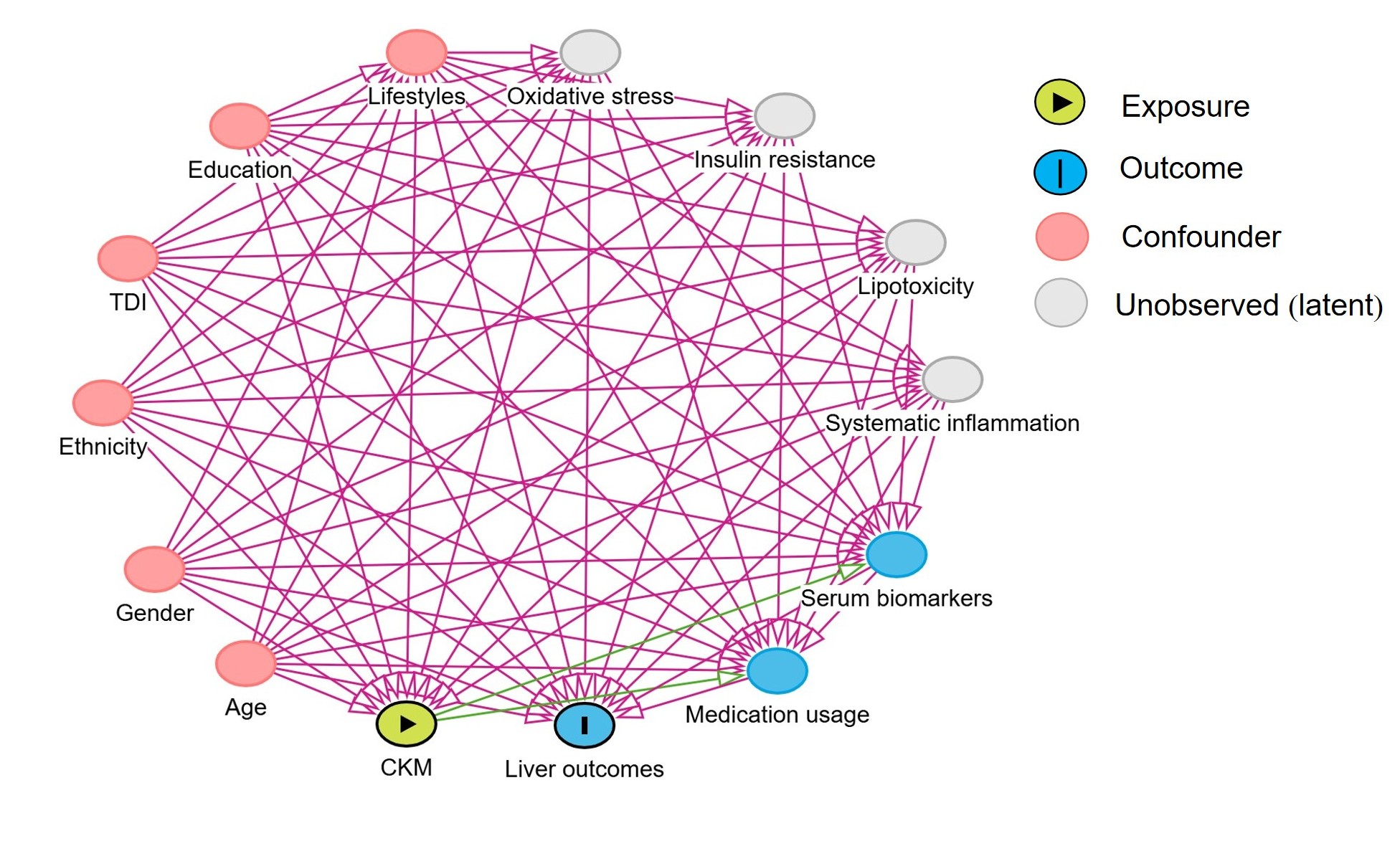


Figure S1. Directed acyclic graph. Lifestyles indicate sleep duration, physical activity, diet, smoking and drinking status.Serum biomarkers refer to ALT/AST, Apo A/B, CRP, and albumin. Medication use indicates antihypertensives, hypoglycemics and so on.


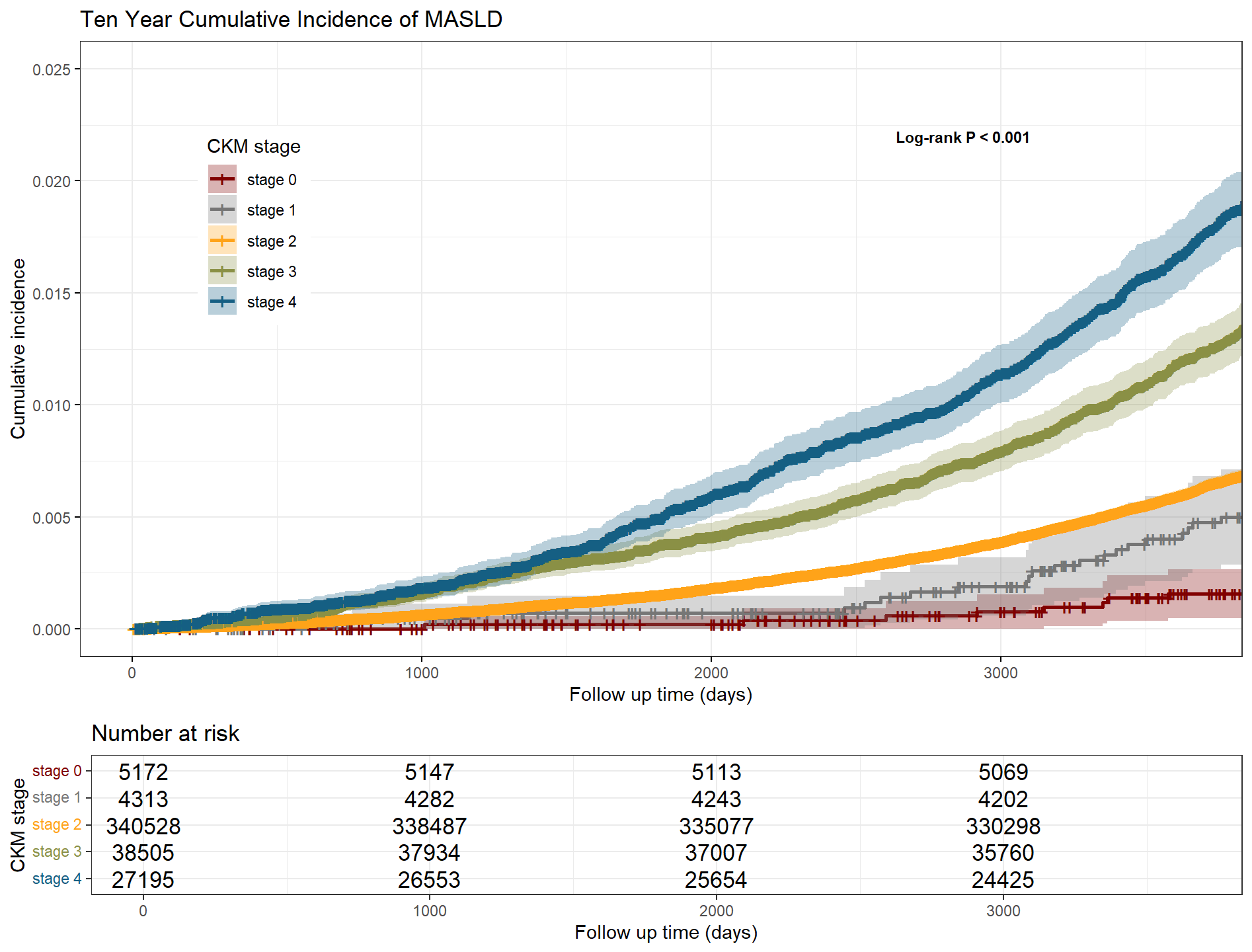


Figure S2 Ten year cumulative incidence of MASLD by CKM stages


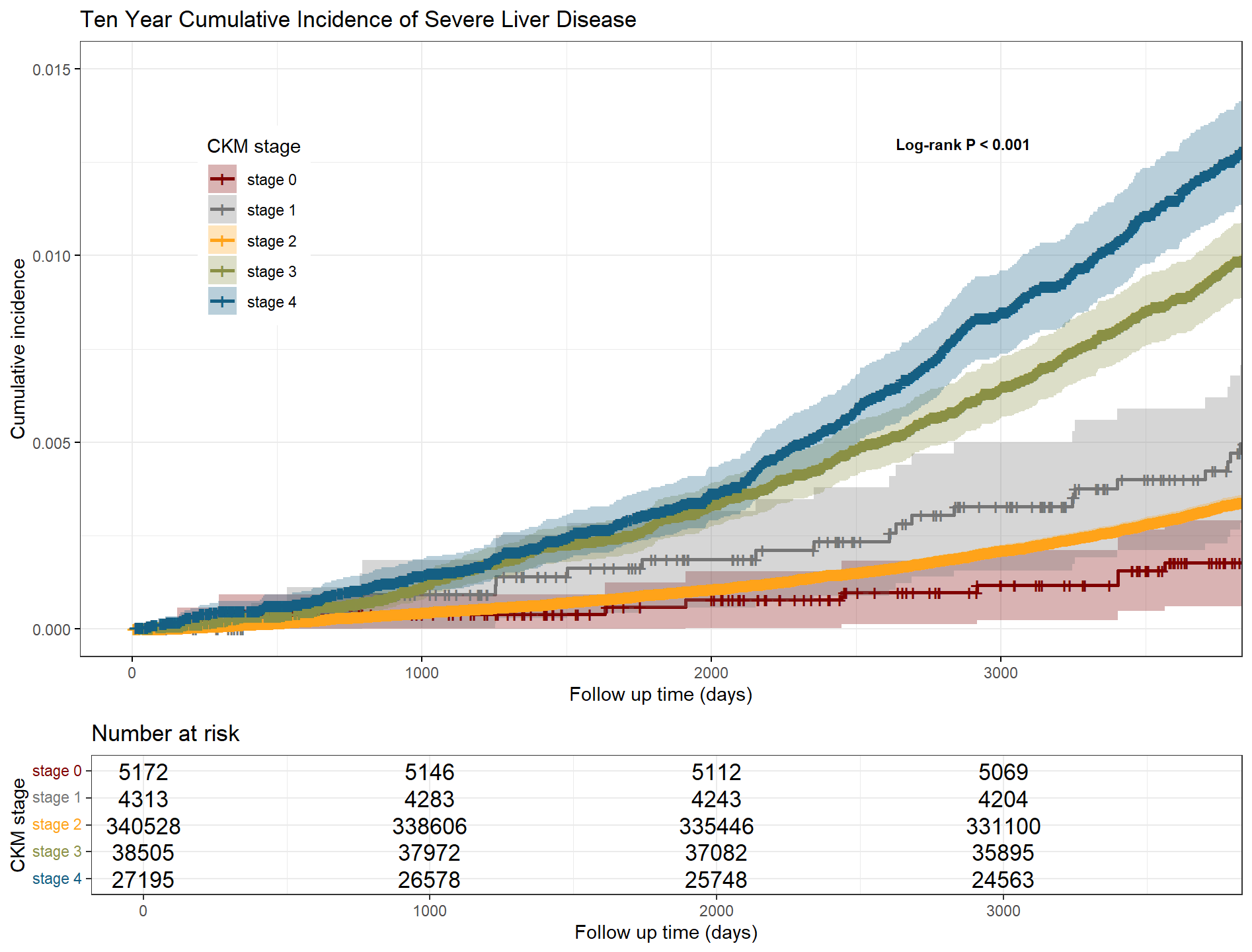


Figure S3 Ten year cumulative incidence of severe liver disease by CKM stages


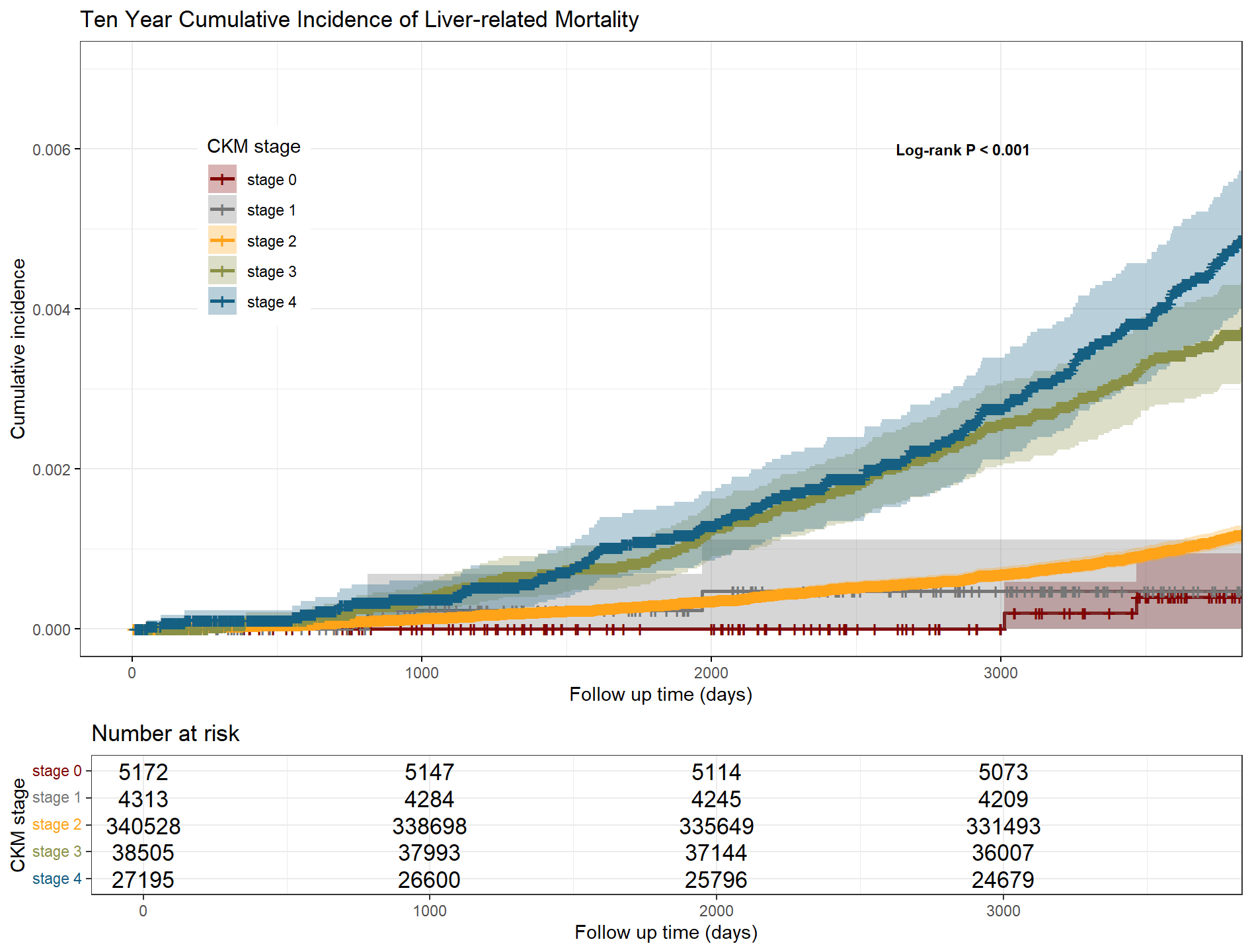


Figure S4 Ten year cumulative incidence of liver-related death by CKM stages


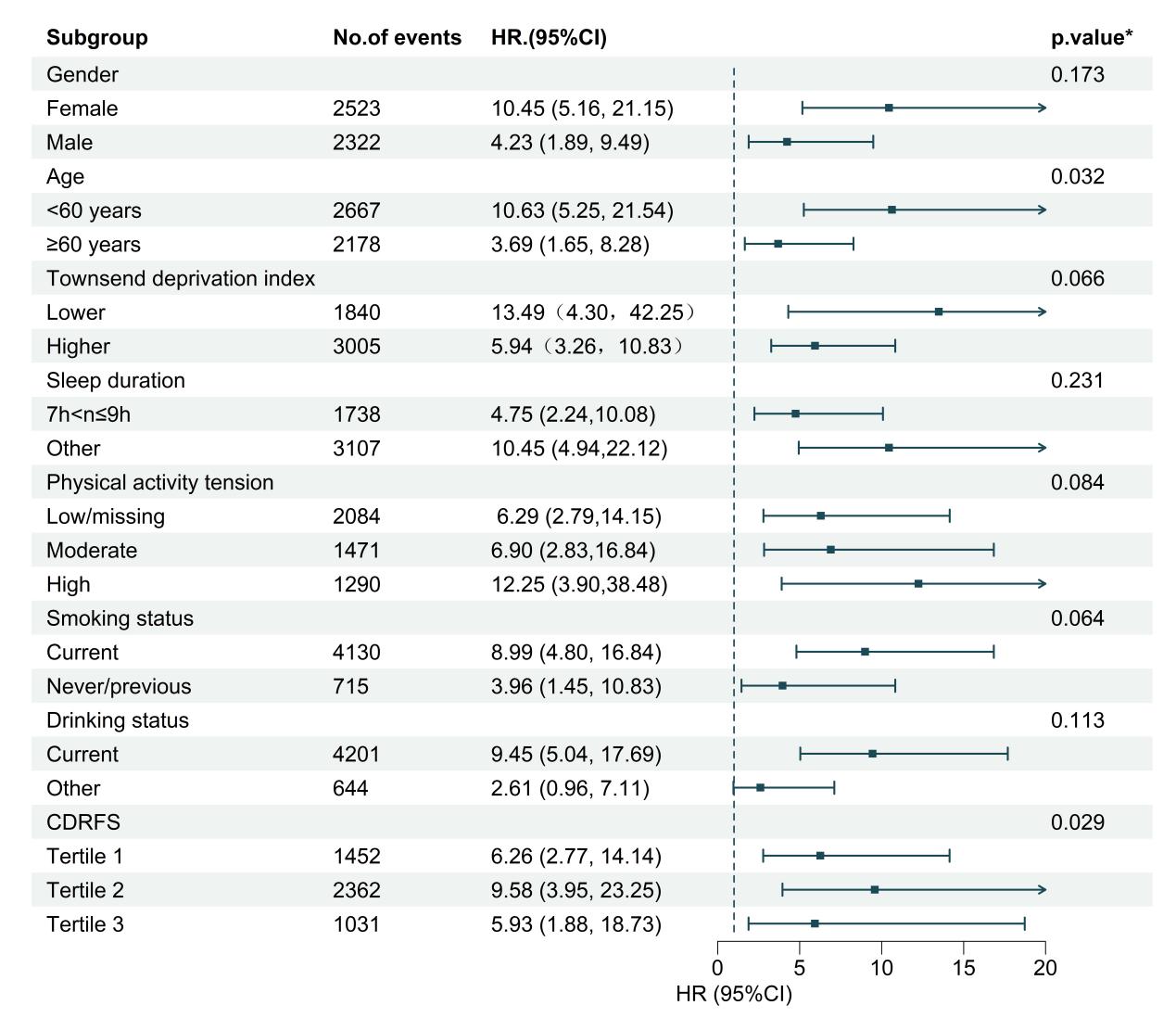


Figure S5. Stratified analyses of the associations of CKM stage with risk of MASLD. Multiplicative interactions were calculated by inserting the product terms into the multivariable Cox model 2, and each stratified variable itself was not included in the model. * p.value refer to p for interaction. HR, hazard ratio; MASLD, metabolic dysfunction-associated steatotic liver disease. CDFRS, cumulative dietary risk factor score; TDI, Townsend deprivation index;


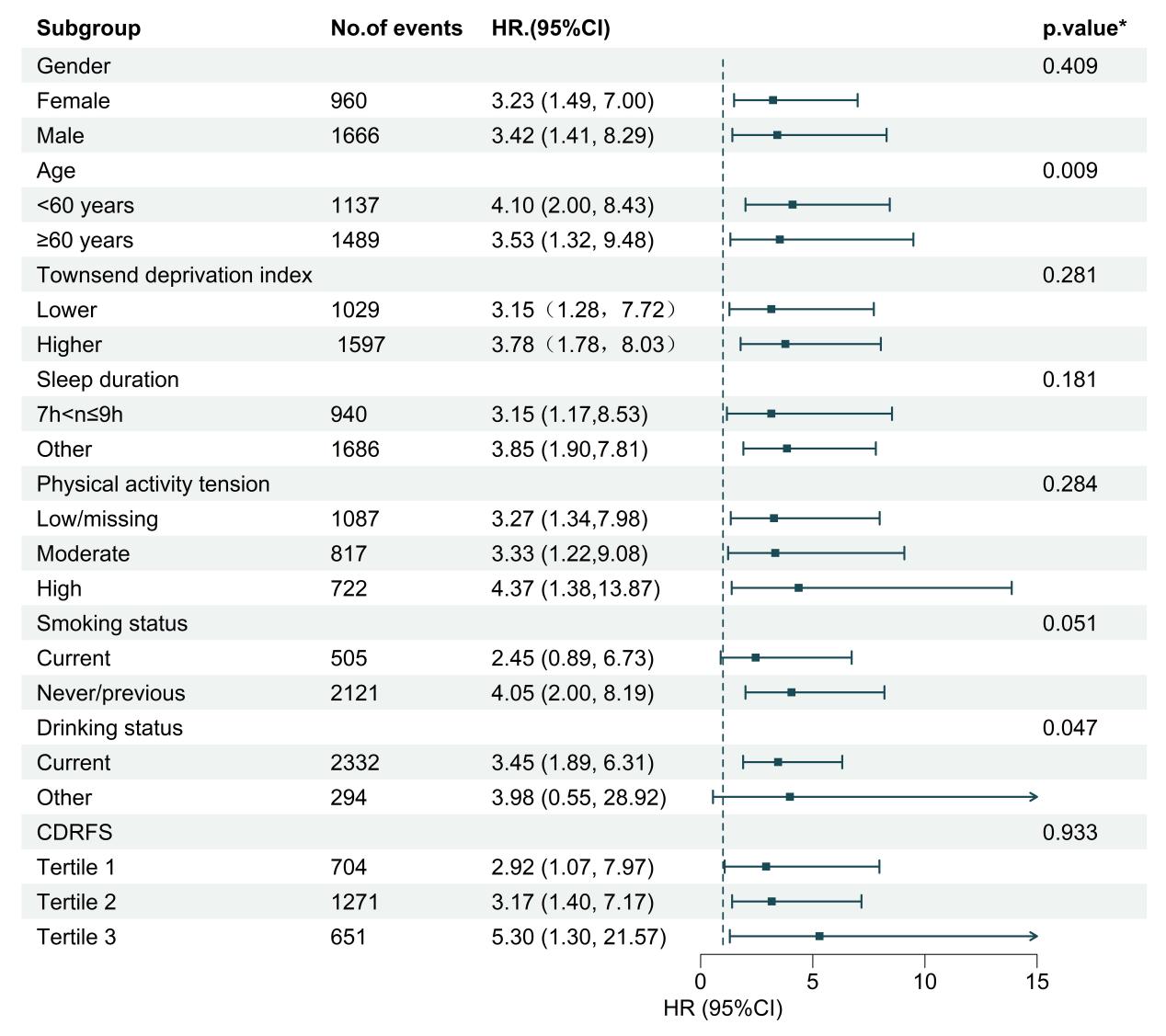


Figure S6. Stratified analyses of the associations of CKM stage with risk of severe liver disease. Multiplicative interactions were calculated by inserting the product terms into the multivariable Cox model 2, and each stratified variable itself was not included in the model. *p.value refer to p for interaction. HR, hazard ratio; CDFRS, cumulative dietary risk factor score; TDI, Townsend deprivation index;


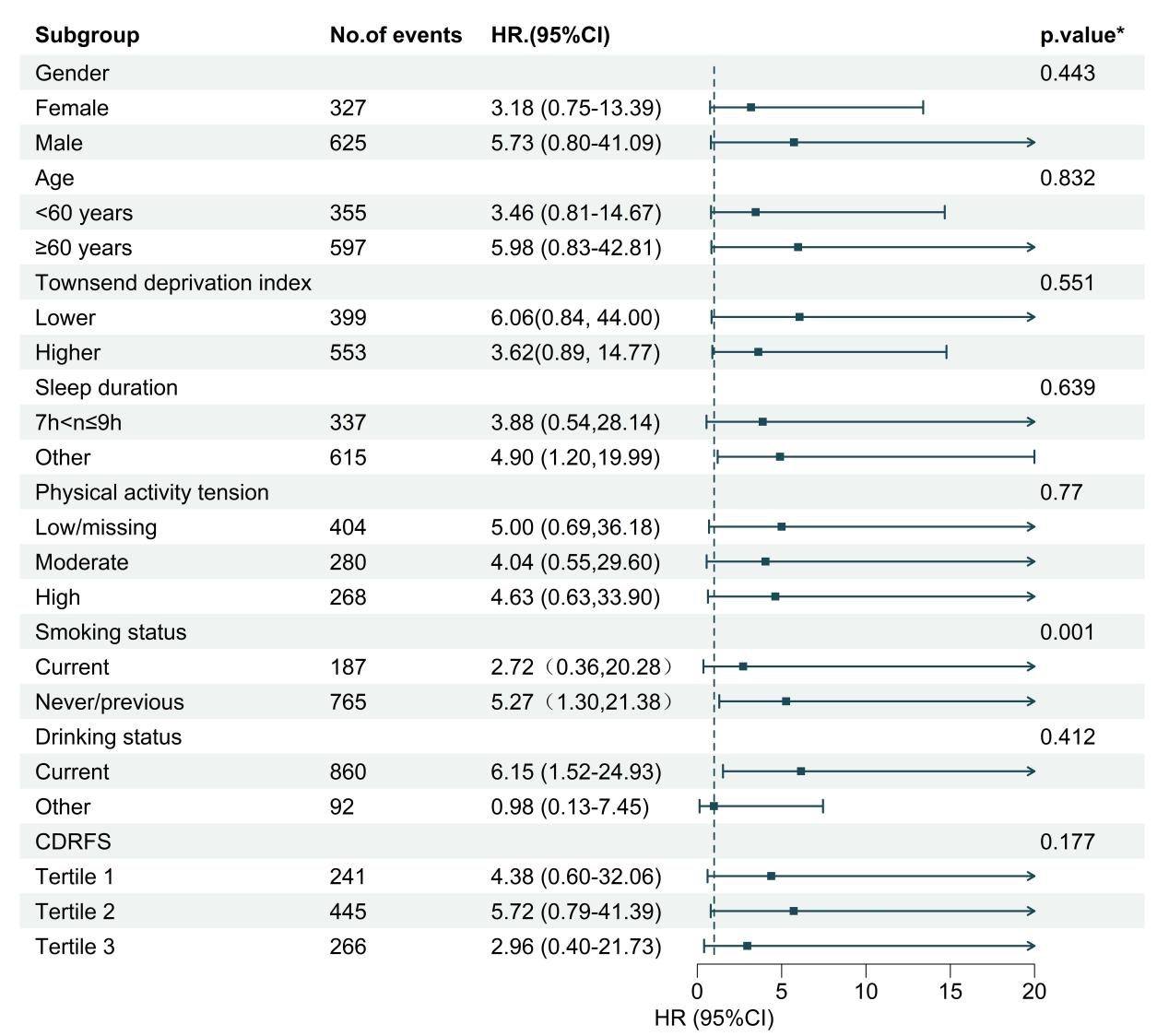


Figure S7. Stratified analyses of the associations of CKM stage with risk of liver-specific mortality. Multiplicative interactions were calculated by inserting the product terms into the multivariable Cox model 2, and each stratified variable itself was not included in the model. *p.value refer to p for interaction. HR, hazard ratio; CDFRS, cumulative dietary risk factor score; TDI, Townsend deprivation index;
